# The Fusiform Face Area Plays a Greater Role in Holistic Processing for Own-Race Faces Than Other-Race Faces

**DOI:** 10.3389/fnhum.2018.00220

**Published:** 2018-06-01

**Authors:** Guifei Zhou, Jiangang Liu, Naiqi G. Xiao, Si Jia Wu, Hong Li, Kang Lee

**Affiliations:** ^1^School of Computer and Information Technology, Beijing Jiaotong University Beijing, China; ^2^Department of Psychology, Princeton University Princeton, NJ, United States; ^3^Dr. Eric Jackman Institute of Child Study, University of Toronto Toronto, ON, Canada; ^4^College of Psychology and Sociology, Shenzhen University Shenzhen, China; ^5^Center for Language and Brain, Shenzhen Institute of Neuroscience Shenzhen, China; ^6^Shenzhen Key Laboratory of Affective and Social Cognitive Science, Shenzhen University Shenzhen, China; ^7^Department of Psychology, Zhejiang Normal University Jinhua, China

**Keywords:** other-race effect, composite face effect, fMRI, FFA, holistic face processing

## Abstract

Own-race faces are recognized more effectively than other-race faces. This phenomenon is referred to as other-race effect (ORE). Existing behavioral evidence suggests that one of the possible causes of ORE is that own-race faces are processed more holistically than other-race faces. However, little is known about whether such differences in processing also produce distinctive neural responses in the cortical face processing network. To bridge this gap, the present study used fMRI methodology and the composite face paradigm to examine the response patterns of the traditional face-preferential cortical areas (i.e., the bilateral fusiform face areas [FFA] and the bilateral occipital face areas [OFA]) elicited by own-race faces and other-race faces. We found that the right FFA exhibited a neural composite face effect only for own-race faces but not for other-race faces, even with the absence of the race-related difference in behavior composite face effect. These findings suggest that the right FFA plays a greater role in holistic processing of individual own-race faces than other-race faces. They also suggest that the neural composite effect observed in the right FFA is not the exact neural counterpart of the behavioral face composite effect. The findings of the present study revealed that, along the pathway of the bottom-up face processing, own-race faces and other-race faces presented the holistic processing difference as early as when they were processed in the right FFA.

## Introduction

People recognize own-race faces faster and more accurately than other-race faces and this behavioral phenomenon is referred to as the other-race effect (ORE; Meissner and Brigham, 2001; Hugenberg et al., 2010; Young et al., 2012). It has been suggested that the behavioral ORE is due to the fact that own-race faces and other-race faces are processed in different manners (for a review see Hugenberg et al., 2010). In particular, existing behavioral evidence suggests that one major difference in processing is that own-race faces are processed more holistically than other-race faces (e.g., Tanaka et al., 2004; Michel et al., 2006). However, little is known about whether such differences in processing also produce distinctive neural responses in the cortical face processing network. The present study aimed to bridge this significant gap by specifically focusing on whether holistic processing of own- and other-race faces produces differential neural responses in the core face processing areas in the ventral occipitotemporal cortex.

Behavioral holistic face processing studies have used a well-established method called the composite face effect paradigm. The composite face effect refers to the phenomenon that when participants are asked to recognize the top or bottom halves of a composite face with the top and bottom halves coming from different individuals, they recognize more accurately when the top and bottom halves of the faces are misaligned than when they are aligned (Young et al., 1987; for review, see Rossion, 2013). This difference in recognition accuracy is due to the fact that the top and bottom halves of the face stimuli are processed holistically by fusing the two halves as a whole. As a result, the top half or the bottom half cannot be readily recognized by itself (Rossion, 2013).

Some behavior studies have also revealed that the size of the composite face effect is closely related to one’s ability to recognize face identities (e.g., Richler et al., 2011; Wang et al., 2012): the greater the effect, the better one’s face recognition memory. The composite face effect has also been found with non-face objects (e.g., Greebles) with which participants have visual recognition expertise (Gauthier et al., 1998; Gauthier and Tarr, 2002). It has thus been suggested that holistic face processing may be engendered by one’s visual processing expertise (the holistic expertise hypothesis).

Given the fact that individuals tend to have greater expertise at processing own-race faces than other-race ones, one should expect own-race faces to be processed more holistically than other-race faces, resulting in greater composite effects. Indeed, Michel et al. (2006) used the composite effect paradigm and compared the sizes of the composite effects when participants recognized own- and other-race faces. They found that the recognition of own-race faces exhibited greater composite face effects than that of other-race faces, supporting the holistic expertise hypothesis. No similar neural imaging studies have been conducted. Thus, it is entirely unclear as to whether own- and other-race faces produce differential neural composite effects similar to the behavioral effects.

A recent functional neuroimaging study by Schiltz et al. (2010) on own-race face processing with an event-related adaptation paradigm suggests that the composite face effect engenders specific neural responses in the core face processing network in the ventral occipitotemporal cortex. Specifically, the researchers focused on the face-preferential cortical regions (i.e., the bilateral fusiform face area; [FFA, Kanwisher et al., 1997] and the bilateral occipital area [OFA, Gauthier et al., 2000]). They found that the paired faces with the same top haves but different bottom haves elicited a release of adaptation of fMRI responses of the right FFA for aligned faces but not for misaligned faces. Further, the size of this release of adaptation was equal to that elicited by the paired faces with completely different identities. These findings suggested that the recognition of top haves of faces were automatically influenced by the bottom haves of faces for aligned faces, but not for misaligned faces. Such difference between aligned faces and misaligned faces in fMRI response pattern was similar to that in behavior performance, suggesting a neural composite face effect in the right FFA (Schiltz et al., 2010). These findings suggested that the right FFA may be indeed involved in holistic face processing. However, because only own-race face stimuli were used in the study of Schiltz et al. (2010), it is yet unclear what role the FFA plays in the holistic processing of own- vs. other-race faces.

Although no neuroimaging studies have directly tested the holistic expertise hypothesis, some existing evidence suggests that this hypothesis may be true at the neural level. It is well established that for own-race faces, the FFAs, particularly the right one, are closely associated with the recognition of face identity (Kanwisher et al., 1997; Kanwisher and Yovel, 2006), and play a key role in the neural network of face processing (Haxby et al., 2000; Fairhall and Ishai, 2007). Thus, most of the existing functional neuroimaging studies on ORE have focused on differences in the FFA responses to own- vs. other-race faces. Evidence to date suggests that own- and other-race faces indeed engender differential responses in the FFAs (Golby et al., 2001; Kim et al., 2006; Feng et al., 2011; Liu et al., 2015). However, due to the fact that none of the studies specifically manipulated holistic processing, it is unclear as to whether holistic processing would engage the responses of FFAs differentially to own- vs. other-race faces.

The present study aimed to bridge this significant gap in the literature. We specifically examined whether the FFAs respond similarly or differentially when participants are engaged in holistic processing of own- and other-race faces, using the composite face effect paradigm. To this end, we adapted the experimental paradigm of Schiltz et al. (2010) by adding other-race faces in the design such that we could compare the neural composite face effects elicited by own-race faces to those by other-race faces. If the holistic expertise hypothesis is true, own-race faces should be processed more holistically than other-race face, thus eliciting greater neural composite face effects in the FFA (particularly the right hemisphere) than other-race faces. Otherwise, holistic processing of own- and other-race faces should elicit similar composite face effects in the FFAs.

## Materials and Methods

### Participants

Forty healthy, right-handed Han Chinese adults (18 males; Mean Age ± SD: 21.4 ± 1.7 years) with normal or corrected-to-normal vision participated. As reported by these participants, none of them had the experience of ever working or living with Caucasian or being engaged into the work that needed to directly contact with Caucasian or Caucasian face images. A written informed consent was signed by all participants prior to the experiment. The present study was approved by the Southwest University research ethics committee.

### Stimuli and Experimental Procedures

#### Stimuli

The experimental paradigm of the present study was adapted from Schiltz et al. (2010). Our experiments included two fMRI periods: the event-related composite face recognition period and the block-design functional localizer period.

**Figure 1:**
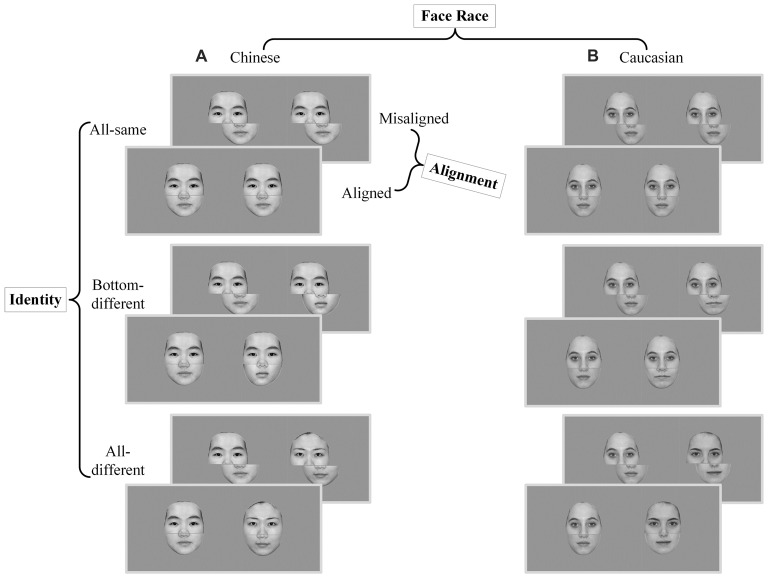
Experimental stimuli and conditions of composite face recognition period. The composite face recognition period was a 2 × 2 × 3 factorial within-participant design, namely *face race* (*Chinese* vs. *Caucasian*) by *alignment* (*aligned* vs. *misaligned*) by *identity* (*all-same* vs. *bottom-different* vs. *all-different*). Each trial included a pair of faces presented in sequence, both of which were either Chinese faces **(A)** or Caucasian faces **(B)** and were either aligned or misaligned. *All-same*: the pair of faces of a trial have the same identities; *Bottom-different*: the pair of faces of a trial have the same top halves but different bottom halves; *All-different*: the pair of faces of a trial have completely different identities.

The composited face images (about 80%) of the present study were based on the Chinese and Caucasian face images used in a behavioral study of ORE (Ge et al., 2009). In this study, these face images were used as experimental stimuli as own- vs. other-race faces for Chinese and Caucasian participants. Reliable OREs were found for both Caucasian and Chinese participants. Further, there were few of differences in the face configural information between own-race faces and other-race faces (for more details see Part I of Supplementary Materials). In the present study, all face stimuli were converted into gray-scale images with a resolution of 1024 × 768 pixels. Similar to the stimulus preparation procedure used in Ge et al. (2009), the low-level visual properties of the images of the present study such as luminance and contrast were balanced using a standard MATLAB procedure (SHINE, Willenbockel et al., 2010). Further, the external features of each face (e.g., ears and hair style) were removed. To create composite faces, a gray gap with a width of 3-pixels across the tip of the nose was used to split the face into the top half and the bottom half (see Figure 1). The spanned visual angle of image stimuli was about 12.7° by 12.3°.

#### Experimental Procedures

As mentioned above, a neural composite face effect can be indicated by a release of adaptation of fMRI response within a cortical region. More specifically, it should meet three criteria. First, this cortical region itself should present the adaptation of fMRI responses elicited by the repetition of face identities. In other word, the fMRI responses of this cortical region should be stronger for the paired faces with completely different identities than those with the same identities for both aligned faces and misaligned faces. Meeting this criterion means that this cortical region is sensitive to the face identities. Second, the fMRI response should be stronger for the paired faces with the same top- but different bottom-haves of faces than for the paired faces with the same identities with the response increase significant for aligned faces, but not for misaligned faces. Third, for aligned faces, such response increase should be equal to that elicited by the paired faces with completely different identities minus the paired faces with the same identities.

According to these requirements, the composite face recognition period was a 2 × 2 × 3 factorial within-participant design, namely *face race* (*Chinese* vs. *Caucasian*) by *alignment* (*aligned* vs. *misaligned*) by *identity* (*all-same* vs. *bottom-different* vs. *all-different*; see Figure 1). For the sake of convenience, the names of these conditions will be referred to by their respective *italic* types in the present study. For *aligned*, the top and bottom halves were processed to be precisely aligned to each other, whereas for *misaligned*, the top and bottom halves were processed to be slightly misaligned to each other. The misaligned face stimuli were created by moving the bottom halves to the right to an extent that the right edges of the top half of nose meet the left edges of the bottom half of nose. For *all-same* trials, both the top and bottom halves of the paired faces were the same; for *bottom-different* trials, only the top halves of the paired faces were the same, but the bottom halves were different; for *all-different* trials, the paired faces had completely different identities.

**Figure 2:**
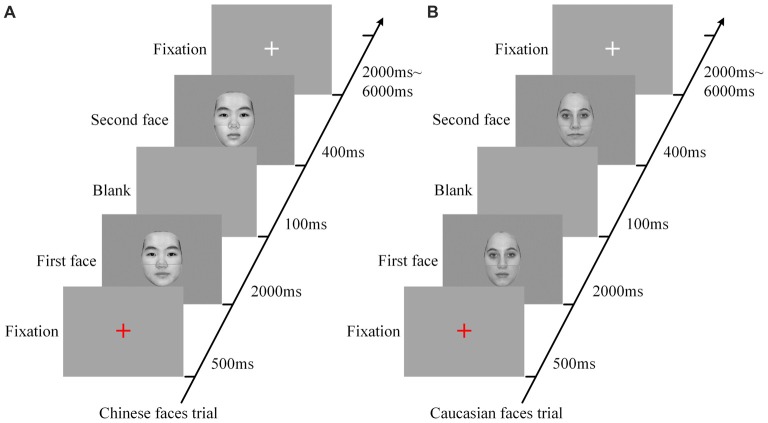
The procedures of experimental trials for Chinese face condition **(A)** and Caucasian face condition **(B)**. Each trial, including a pair of faces presented in sequence, began with a red fixation as a prompt (500 ms) followed by the first face (2000 ms), and then included a blank (100 ms) followed by the second face (400 ms), and ended up with a white fixation for a random duration of 2000–6000 ms. In each trial, the participants were instructed to focus on the top halves of the two faces and make a response as to whether the identities of these two faces are identical. ms: Millisecond.

Six scanning sessions were included in the composite face recognition period, three for the Chinese faces and three for the Caucasian faces. Each of the sessions for the Chinese face condition that included only Chinese faces contained 48 trials with random intervals from 2000 ms to 6000 ms. As shown in Figure 2, each trial began with a red fixation as a prompt (500 ms), and then included a pair of faces presented in sequence (2000 ms for the first face, and 400 ms for the second face) with an interval of a blank screen of 100 ms between them. The relatively long presentation of the first face (2000 ms) was to ensure that the participants could successfully remember this face. As to the short length of the presentation of the second face, as demonstrated by our advance behavior test (for detail see Part IV of Supplementary Materials), the presentation of 150 ms was sufficient to lead to an accuracy rate of about 80% and a behavior composite effect. Additionally, taking into account of the influence of the poor environment within the MRI scanner as well as the limitation of fMRI scanning time, we set the length of the presentation of the second faces as 400 ms. As demonstrated by the results of the present study, these parameters were appropriate for the experimental deign of the present study. The participants were required to focus on the top halves of these two faces, and to make a decision as to whether they were identical via button presses (counterbalanced across participants). These 48 pairs of faces consisted of a 2 × 3 factorial within-participant design (eight trials for each cell), namely *alignment* (*aligned* vs. *misaligned*) by *identity* (*all-same* vs. *bottom-different* vs. *all-different*). All trials in each of the three sessions of the Chinese face condition were presented in random order. Each fMRI scanning session began with a 4 s central fixation period to calm participants and ended with a 10 s central fixation period to allow the BOLD signal elicited by the last trial to return to baseline.

The experimental design of the sessions for the Caucasian face condition was the same as that of the Chinese face condition except that the Chinese faces were replaced by Caucasian faces. The Chinese face sessions and the Caucasian face sessions were scanned in a counterbalanced order across all participants.

Following the composite face recognition period was the functional localizer period, the aim of which was to identify the traditional cortical areas with preferential responses to faces (i.e., the FFA and the OFA). All stimuli used in this period were gray-scale photos of adults with a resolution of 640 × 480 pixels. All faces (half Chinese and half Caucasian, of which half were male and half were female), had not been seen by participants previously. They were upright and frontal with neutral expressions, and had their original external features (e.g., hair style). The non-face objects were commonly found in daily life such as chairs, glasses and flowers. This period included two localizer sessions. Each session consisted of alternating eight blocks of photos, namely, four blocks of faces (2 for Chinese faces and 2 for Caucasian faces) and four blocks of non-face objects. Each block contained 10 photos, which was presented for 1000 ms followed by an interval of 1000 ms fixation. In each session, the participants were instructed to perform a one-back identity task (two target photos per block) to keep their attention to the stimulus.

As only Chinese participants participated in the present study, we referred to Chinese faces stimuli as own-race faces and Caucasian faces stimuli as other-race faces in the present study, respectively.

### fMRI Data Acquisition

MRI data in the present study were acquired using a 3.0 Tesla MRI scanner (Siemens Trio a Tim, Germany) at Southwest University (Chongqing China). The participants during both the composite face recognition period and the functional localizer period were scanned using standard EPI sequences with a 12-channel head coil (TR = 2000 ms, TE = 30 ms, FOV = 220 × 220 mm^2^, flip angle = 90°, matrix = 64 × 64 mm^2^, voxel size = 3.44 × 3.44 × 4 mm^3^, number of slices = 32). A total of 165 and 139 whole brain T2*-weighted axial images (phase encode direction: AP) were acquired for each run of the composite face recognition period and each run of the functional localizer period, respectively. Additionally, for each participant, 3D T1-weighted structural images were acquired using a magnetization prepared rapid acquisition gradient echo sequence (voxel size: 1 × 1 × 1 mm^3^, FOV = 256 × 256 mm^2^).

### fMRI Data Analysis

The first two fMRI volumes of each session were discarded. Analysis for the remaining data, including preprocessing and statistical, were mainly implemented using the Statistical Parametric Mapping software (SPM12, Wellcome Trust Centre for Neuroimaging, London, UK^1^, Friston et al., 1995). During the preprocessing, after slice-timing corrections, spatial realignment and co-registration to the T1-weighted structural images, all fMRI scans were spatially normalized to the MNI (Montreal Neurological Institute) template and were resampled to 2 × 2 × 2 mm^3^ voxels. Then, all these data were spatially smoothed using an isotropic 6 mm full-width-half-maximal.

After preprocessing, the fMRI data obtained from the composite face recognition period and the functional localizer period were analyzed separately. For the fMRI data of the composite face recognition period of each participant, we constructed a general linear model (GLM) with 12 regressors indicating the 12 experimental conditions, which were produced by the 2 × 2 × 3 factorial within-participant design, namely *face race* (*own-race* vs. *other-race*) by *alignment* (*aligned* vs. *misaligned*) by *identity* (*all-same* vs. *bottom-different* vs. *all-different*; Figure 1).

Each regressor was produced by the convolution of the canonical hemodynamic response function (HRF) and a delta function corresponding to the onset sequence of the first face presentation in the trials of each experimental condition. Movement parameters were also included as the regressors of GLM to account for residual effects resulting from movement. All fMRI sessions were high-pass filtered (highpass filter = 128 s) in order to remove low-frequency noise such as scanner drift (Friston et al., 1995). After the estimation of the parameters of GLM, we obtained 12 whole brain maps for the 12 experimental conditions, respectively. Then the whole brain group analysis across all participants was performed on these 12 whole brain maps using a conjunction analysis separately for own-race faces and other-race faces to identify the brain regions that present a neural composite face effect (Goh et al., 2004).

For the fMRI data of the functional localizer sessions of each participant, we also constructed the same GLM as that for the composite face recognition period except the former only included three regressors corresponding to their three experimental conditions, respectively (own-race faces, other-race faces and non-face objects). For each participant, the face-preferential cortical areas (i.e., the bilateral FFAs and the bilateral OFAs) were identified using contrast of ([own- and other-race faces] minus non-face objects) as the regions of interest (ROI). The basic statistical threshold of ROI extraction was *p* < 0.0001(uncorrected) and *Cluster* ≥ 10. However, taking into account the great individual differences in the size and intensity of these face-preferential cortical areas and in order to identify the ROIs across participants as many as possible, we loosened this threshold to *p* < 0.001 (uncorrected) and *Cluster* ≥ 10. If not, then we continued to loosen it to *p* < 0.005 (uncorrected) and *Cluster* ≥ 10 or even *p* < 0.01 (uncorrected) and *Cluster* ≥ 10 (Liu et al., 2015). Additionally, for some extremely large activated areas that even extended to other cortical regions, the ROI was extracted using a sphere of customized radius with the center at the activation peak.

### Whole Brain Conjunction Analysis

In the present study, the neural composite face effect was indicated by a release of adaptation of fMRI response, and it should meet three criteria described above. Thus, to identify the brain region presenting neural composite face effect, we used the conjunction analysis in the whole brain analysis separately for own-race faces and other-race faces (Goh et al., 2004). This whole brain conjunction analysis was performed in the group analysis across all participants. To this end, taking an example of own-race faces, we needed to construct some contrasts according to the criteria of neural composite face effect above mentioned. The first contrast was *aligned all-different* minus *aligned all-same* (C1). The second contrast was *misaligned all-different* minus *misaligned all-same* (C2). The conjunct activation of C1 and C2 met the first criterion of neural composite face effect. The third contrast was *aligned bottom-different* minus *aligned all-same* (C3). The fourth contrast was (*aligned bottom-different* minus *aligned all-same*) minus (*misaligned bottom-different* minus *mi*s*aligned all-same*; C4). The conjunct activation of C3 and C4 met the second criterion of neural composite face effect. The fifth contrast was *aligned all-different* minus *aligned bottom-different* (C5). Different from the C1~C4, the inactivation of C5 met the third criterion of neural composite face effect. Thus, the activation of C1&C2&C3&C4 and the inactivation of C5 indicated a neural composite face effect for *own-race* faces. To perform this voxel-wise whole brain conjunction analysis in the present study, we first selected the brain regions that did not show activation of C5 (*p* < 0.001) as a mask. Then, in this mask we performed a voxel-wise conjunction analysis of C1&C2&C3&C4 (*p* < 0.001 uncorrected, *Cluster* ≥ 10) to identify the conjunctly activated brain regions. We also performed the same conjunction analysis for other-race faces as that for own-race faces using the whole brain maps of experimental conditions of other-race faces.

### ROI Analysis

For each of the bilateral FFAs and the bilateral OFAs, the percent signal change (PSC) of fMRI responses elicited by each of the experimental conditions during the composite face recognition period was calculated using MarsBar software (Brett et al., 2002). First, within each ROI, a 2 × 2 × 3 repeated-measure three-way analysis of variance (ANOVA), namely *face race* (*own-race* vs. *other-race*) by *alignment* (*aligned* vs. *misaligned*) by *identity* (*all-same* vs. *bottom-different* vs. *all-different*) was performed on the PSC.

Second, if the interaction effect of *face race* by* alignment* by* identity* was significant, we will perform statistical analyses on the PSCs for own-race faces and other-race faces separately to explore the crucial effect of the interaction further. For example, within the right FFA, a 2 × 3 repeated measure two-way ANOVA, namely *alignment* (*aligned* vs. *misaligned*) by *identity* (*all-same* vs. *bottom-different* vs. *all-different*) was performed on the PSC elicited by own-race faces. If the interaction effect of *alignment* by *identity* was significant, the *post hoc* tests will be performed. Particularly, according to the criteria of neural composite effect, we first examined whether a normal release of adaptation was generated by contrast of *all-different* vs. *all-same*. We then examined whether there was another release of adaptation elicited by contrast of *bottom-different* vs. *all-same*. Finally, we examined whether the release of adaptation elicited by *bottom-different* was equal to a normal release of adaptation by contrast of (*all-different* minus *all-same*) vs. (*bottom-different* minus* all-same)*.

## Results

### Behavior Results

Table 1 showed the mean accuracy rates for each of the 12 experimental conditions. In the present study, as the distribution of the accuracy rate is binomial rather than normal, a generalized estimating equations approach instead of ANOVA analysis was performed on the accuracy rate with the *face race* (*own-race* vs. *other-race*), *alignment* (*aligned* vs. *misaligned*), and *identity* (*all-same* vs. *bottom-different* vs. *all-different*) as the within-subject variables, and the binomial response with a logit link function as the response.

**Table 1:** Accuracy rate and correct response time (RT) of composite own- and other-race faces task.

		All-same		Bottom-different		All-different	
		Aligned	Misaligned	Aligned	Misaligned	Aligned	Misaligned
*Accuracy rate*							
Own-race faces	Mean	0.94	0.94	0.60	0.91	0.95	0.94
	SD	0.11	0.11	0.25	0.14	0.13	0.11
							
Other-race faces	Mean	0.96	0.95	0.64	0.93	0.98	0.96
	SD	0.05	0.07	0.26	0.07	0.04	0.04
*Correct response time (ms)*						
Own-race faces	Mean	835	833	983	853	859	886
	SD	199	177	249	177	161	169
							
Other-race faces	Mean	832	843	1011	861	855	876
	SD	178	182	259	178	169	159

*SD indicates standard deviation*.

A generalized estimating equations analysis (*face race* × *alignment* × *identity*) on these accuracy rates revealed a significant main effect of *identity* (*Wald* $x_{\left(2\right)}^{2}$ = 121.441, *p* < 0.05) and that of *alignment* (*Wald* $x_{\left(1\right)}^{2}$ = 23.381, *p* < 0.05). The significant main effect of *alignment* was due to the fact that the participants recognized faces less accurately for *aligned* faces than for *misaligned* faces (*p* < 0.05), thus replicating the robust behavioral face composite effect. However, neither the interaction effect of *face race* × *alignment* × *identity* (*Wald* $x_{\left(2\right)}^{2}$ = 0.617, *p* = 0.735) nor the main effect of *face race* (*Wald* $x_{\left(1\right)}^{2}$ = 3.336, *p* = 0.068) was significant. These results suggested that the face composite effects were not significantly different from each other for own- vs. other-race faces.

**Figure 3:**
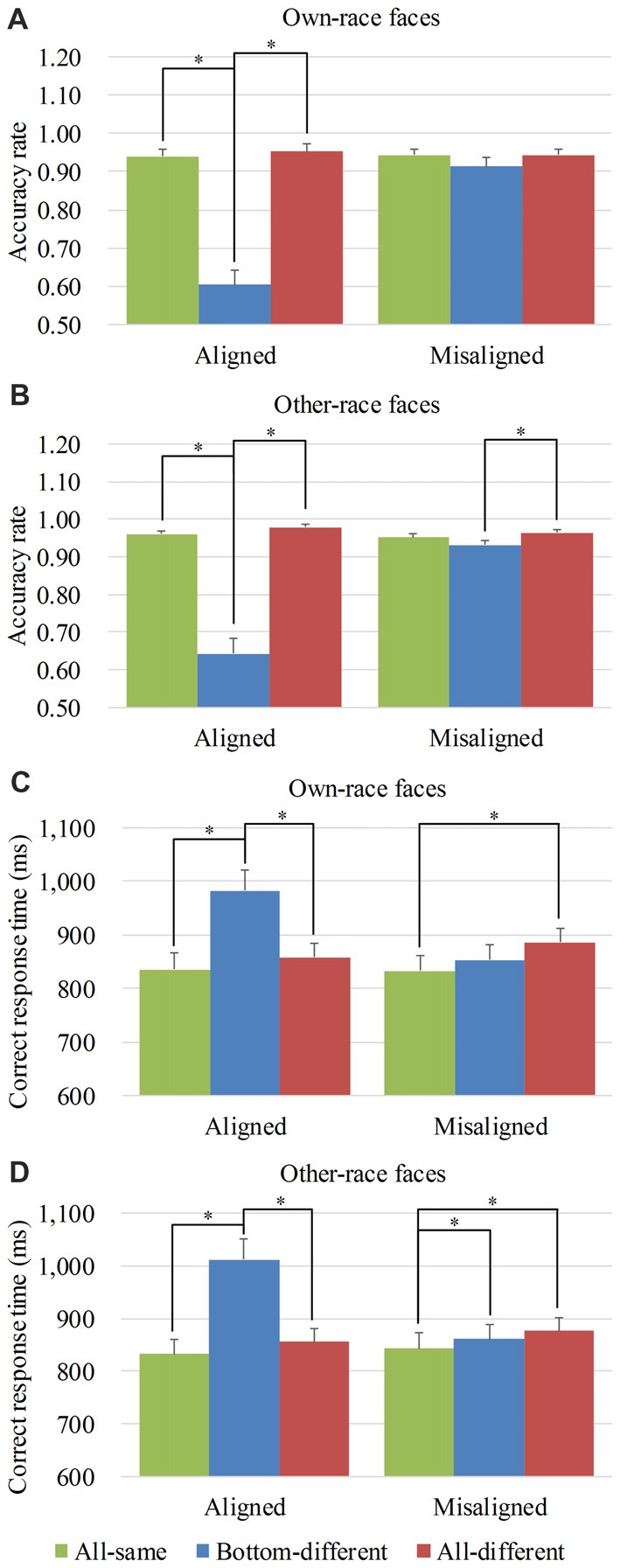
Behavior performance of composite face recognition period. Figure showed the mean accuracy rates of own-race faces **(A)** and other-race faces **(B)** as well as the mean correct response time (RT) of own-race faces **(C)** and other-race faces **(D)**. The error bars indicate the standard errors. The “*” indicates *p* < 0.017 (Bonferroni correction).

To explore the performance response to own-race faces and other-race faces, we performed analyses on the mean accuracy rates of own-race faces and other-race faces separately. For the mean accuracy rates of own-race faces, a generalized estimating equations analysis of (*alignment* × *identity*) revealed a significant interaction of *alignment* × *identity* (*Wald* $x_{\left(2\right)}^{2}$ = 56.645, *p* < 0.05), as well as the significant main effects of *identity* (*Wald* $x_{\left(2\right)}^{2}$ = 50.334, *p* < 0.05) and *alignment* (*Wald* $x_{\left(1\right)}^{2}$ = 63.754, *p* < 0.05). Figure 3A shows the mean accuracy rates of own-race faces and Supplementary Figure S3A showed their individual dots plots. As shown in Figure 3A, there was a significant difference among the three levels (i.e., *all-same*, *bottom-different* and *all-different*) only for *aligned* (*Wald* $x_{\left(2\right)}^{2}$ = 81.163, *p* < 0.05), but not for *misaligned* (*Wald* $x_{\left(2\right)}^{2}$ = 5.490, *p* = 0.064). More specifically, participants responded more correctly to *all-same* than to *bottom-different* only for *aligned* (*p* < 0.017 [Bonferroni correction]) but not for *misaligned* (*p* > 0.017 [Bonferroni correction]). Further, this response difference of the *all-same* minus *bottom-different* for *aligned* trials was greater than that for the *misaligned* (*p* < 0.017 [Bonferroni correction]), resulting in a significant interaction of *identity* (*all-same* vs. *bottom-different*) × *alignment* (*aligned* vs. *misaligned*; *Wald* $x_{\left(1\right)}^{2}$ = 55.661, *p* < 0.017 [Bonferroni correction]). Participants responded more correctly to *all-different* than *bottom-different* only for *aligned* (*p* < 0.017 [Bonferroni correction]) but not for *misaligned* (*p* > 0.017 [Bonferroni correction]). Further, this response difference of the *all-different* minus *bottom-different* for *aligned* trials was greater than that for the *misaligned* (*p* < 0.017 [Bonferroni correction]), resulting in a significant interaction of *identity* (*all- different* vs. *bottom-different*) × *alignment* (*aligned* vs. *misaligned*; *Wald* $x_{\left(1\right)}^{2}$ = 34.477, *p* < 0.017 [Bonferroni correction]). In addition, there was no significant difference between *all-different* and *all-same* either for *aligned* (*p* = 0.164) or for *misaligned* (*p* = 0.995).

As for the mean accuracy rates of other-race faces, a generalized estimating equations analysis of (*alignment* × *identity*) revealed a significant interaction of *alignment* × *identity* (*Wald* $x_{\left(2\right)}^{2}$ = 66.217, *p* < 0.05), as well as the significant main effects of *identity* (*Wald* $x_{\left(2\right)}^{2}$ = 93.512, *p* < 0.05) and *alignment* (*Wald* $x_{\left(1\right)}^{2}$ = 5.351, *p* < 0.05). Figure 3B shows the mean accuracy rates of other-race faces and Supplementary Figure S3B showed their individual dots plots. As shown in Figure 3B, there were the significant differences among the three levels (i.e., *all-same*, *bottom-different*, and *all-different*) both for *aligned* (*Wald* $x_{\left(2\right)}^{2}$ = 124.929, *p* < 0.05) and for *misaligned* (*Wald* $x_{\left(2\right)}^{2}$ = 9.586, *p* < 0.05). More specifically, participants responded more correctly to *all-same* than to *bottom-different* only for *aligned* (*p* < 0.017 [Bonferroni correction]) but not for *misaligned* (*p* = 0.080]). Further, this response difference of the *all-same* minus *bottom-different* for *aligned* trials was greater than that for the *misaligned* (*p* < 0.017 [Bonferroni correction]), resulting in a significant interaction of *identity* (*all-same* vs. *bottom-different*) × *alignment* (*aligned* vs. *misaligned*; *Wald* $x_{\left(1\right)}^{2}$ = 46.985, *p* < 0.017 [Bonferroni correction]). Participants responded more correctly to *all-different* than *bottom-different* for *aligned* (*p* < 0.017 [Bonferroni correction]) and for *misaligned* (*p* < 0.017 [Bonferroni correction]). Further, this response difference of the *all-different* minus *bottom-different* for *aligned* trials was greater than that for the *misaligned* (*p* < 0.017 [Bonferroni correction]), resulting in a significant interaction of *identity* (*all- different* vs. *bottom-different*) ×*alignment* (*aligned* vs. *misaligned*; *Wald* $x_{\left(1\right)}^{2}$ = 41.172, *p* < 0.017 [Bonferroni correction]). In addition, there was no significant difference between *all-different* and *all-same* either for *aligned* (*p* = 0.081) or for *misaligned* (*p* = 0.182).

Table 1 also showed the mean correct response time (RT) for each of the 12 experimental conditions. A repeated-measure three-way ANOVA (*face race* × *alignment* × *identity*) on these correct RTs revealed a significant main effect of *identity* (*F*_(2,78)_ = 29.833, *p* < 0.05) and that of *alignment* (*F*_(1,39)_ = 25.979, *p* < 0.05). The significant main effect of *alignment* was due to the fact that the participants recognized faces slower for *aligned* faces than for *misaligned* faces (*t*_(39)_ = 5.097, *p* < 0.05). However, neither the interaction effect of *face race* × *alignment* × *identity* (*F*_(2,78)_ = 1.058, *p* = 0.352) nor the main effect of *face race* (*F*_(1,39)_ = 0.102, *p* = 0.751) was significant. These results suggested that there was no significant difference in the mean correct RT between own-race and other-race faces.

To explore the performance response to own-race faces and other-race faces, we performed analyses on the correct RTs of own-race faces and other-race faces separately. For the correct RTs of own-race faces, a repeated measure two-way ANOVA of (*alignment* × *identity*) revealed a significant interaction of *alignment* × *identity* (*F*_(2,78)_ = 27.261, *p* < 0.05), as well as the significant main effects of *identity* (*F*_(2,78)_ = 17.447, *p* < 0.05) and *alignment* (*F*_(1,39)_ = 11.170, *p* < 0.05). Figure 3C showed the correct RTs of own-race faces and Supplementary Figure S3C showed their individual dots plots. As shown in Figure 3C, there was a significant difference among the three levels (i.e., *all-same*, *bottom-different* and *all-different*) for both *aligned* (*F*_(2,78)_ = 32.130, *p* < 0.05), and *misaligned* (*F*_(2,78)_ = 5.340, *p* < 0.05). More specifically, participants responded more slowly to *bottom-different* than to *all-same* only for *aligned* (*t*_(39)_ = 6.762, *p* < 0.017 [Bonferroni correction]) but not for *misaligned* (*t*_(39)_ = 1.912, *p* = 0.063). Further, this response difference of the *bottom-different* minus *all-same* for *aligned* trials was greater than that for the *misaligned* (*t*_(39)_ = 5.791, *p* < 0.017 [Bonferroni correction]), resulting in a significant interaction of *identity* (*all-same* vs. *bottom-different*) × *alignment* (*aligned* vs. *misaligned*; *F*_(1,39)_ = 33.531, *p* < 0.017 [Bonferroni correction]). In addition, participants showed more RT for *bottom-different* than for *all-different* only when the trials were *aligned* (*t*_(39)_ = 5.422, *p* < 0.017 [Bonferroni correction]), but not when the trials were *misaligned* (*t*_(39)_ = −1.938, *p* = 0.060). Further, this difference of the* all-different* minus* bottom-different* for *aligned* trials was greater than that for *misaligned* (*t*_(39)_ = 6.519, *p* < 0.017 [Bonferroni correction]), resulting in a significant interaction of *identity* (*all-different* vs. *bottom-different*) × *alignment* (*aligned* vs. *misaligned*; *F*_(1,39)_ = 42.498, *p* < 0.017 [Bonferroni correction]). Participants showed more RT to *all-different* trials than to *all-same* trials only for *misaligned* (*t*_(39)_ = 2.636, *p* < 0.017 [Bonferroni correction]), but not for *aligned* (*t*_(39)_ = 1.769, *p* = 0.085). However, we found no significant differences in these response bias between *aligned* and *misaligned* (*t*_(39)_ = −1.349, *p* = 0.185).

As for the correct RTs of other-race faces, a repeated measure two-way ANOVA of (*alignment* × *identity*) revealed a significant interaction of *alignment* ×*identity* (*F*_(2,78)_ = 37.494, *p* < 0.05), as well as the significant main effects of *identity* (*F*_(2,78)_ = 28.456, *p* < 0.05) and *alignment* (*F*_(1,39)_ = 20.427, *p* < 0.05). Figure 3D showed the correct RTs of other-race faces and Supplementary Figure S3D showed their individual dots plots. As shown in Figure 3D, there was a significant difference among the three levels (i.e., *all-same*, *bottom-different* and *all-different*) for both *aligned* (*F*_(2,78)_ = 37.604, *p* < 0.05), and *misaligned* (*F*_(2,78)_ = 5.251, *p* < 0.05). More specifically, participants showed more RT to *bottom-different* than to *all-same* both for *aligned* (*t*_(39)_ = 7.478, *p* < 0.017 [Bonferroni correction]) and for *misaligned* (*t*_(39)_ = 2.681, *p* < 0.017 [Bonferroni correction]). Further, this response difference of the *bottom-different* minus *all-same* for *aligned* trials was greater than that for *misaligned* (*t*_(39)_ = 6.232, *p* < 0.017 [Bonferroni correction]), resulting in a significant interaction of *identity* (*all-same* vs. *bottom-different*) × *alignment* (*aligned* vs. *misaligned*; *F*_(1,39)_ = 38.841, *p* < 0.017 [Bonferroni correction]). The participants showed more RT for *bottom-different* than for *all-different* only when the trials were *aligned* (*t*_(39)_ = 5.578, *p* < 0.017 [Bonferroni correction]), but not when the trials were *misaligned* (*t*_(39)_ = −1.237, *p* = 0.223). Further, this difference of the* bottom-different* minus* all-different* for *aligned* trials was greater than that for *misaligned* (*t*_(39)_ = 6.571, *p* < 0.017 [Bonferroni correction]), resulting in a significant interaction of *identity* (*all-different* vs. *bottom-different*) × *alignment* (*aligned* vs. *misaligned*; *F*_(1,39)_ = 43.181, *p* < 0.017 [Bonferroni correction]). Additionally, participants showed more RT to *all-different* than to *all-same* only for *misaligned* (*t*_(39)_ = 3.024, *p* < 0.017 [Bonferroni correction]), but not for *aligned* (*t*_(39)_ = 1.833, *p* = 0.074). However, we found no significant differences in these response bias between *aligned* and *misaligned* (*t*_(39)_ = −0.863, *p* = 0.393).

### Whole Brain Conjunction Analysis Results

Under the statistical threshold of *p* < 0.001 (uncorrected) and *Cluster* ≥ 10, the whole brain conjunction analysis did not reveal any activation for own-race faces or other-race faces. Taking into account that this threshold may be conservative for the conjunction analysis, we used a less strict threshold of *p* < 0.01 (uncorrected) and *Cluster* ≥ 10. Under this threshold, we did not find any activation for other-race faces but found a patch of cortical areas in the right fusiform gyrus for own-race faces with size of 32 voxels, whose peak location (MNI coordinates [mm]: 42, −48, −16) was highly consistent with that of the right FFA identified by the localizer sessions of the present study (MNI coordinates [mm]: 42 ± 3, −51 ± 6, −18 ± 3, seeing the next part). As described in the next part of the present study, the whole brain conjunction analysis revealed the same findings as those revealed by ROI analysis. Thus, the results of the whole brain analysis will not be discussed further.

### ROI Analysis Results

**Figure 4:**
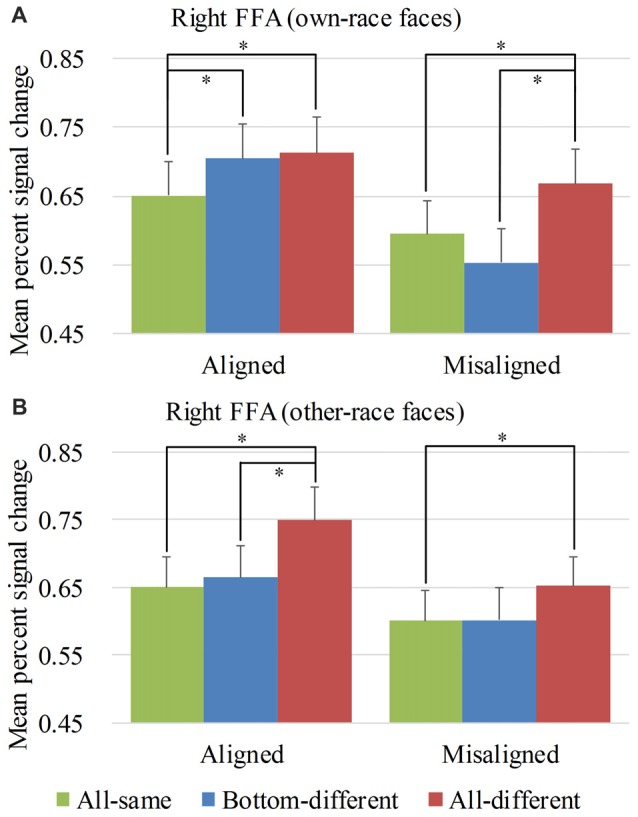
Mean percent signal change (PSC) of the right fusiform face areas (FFA). Mean PSC of the right FFA elicited by each of the conditions during composite face recognition period for own-race faces **(A)** and other-race faces **(B)**. The error bars indicate the standard errors. The “*” indicates *p* < 0.017 (Bonferroni correction).

**Figure 5:**
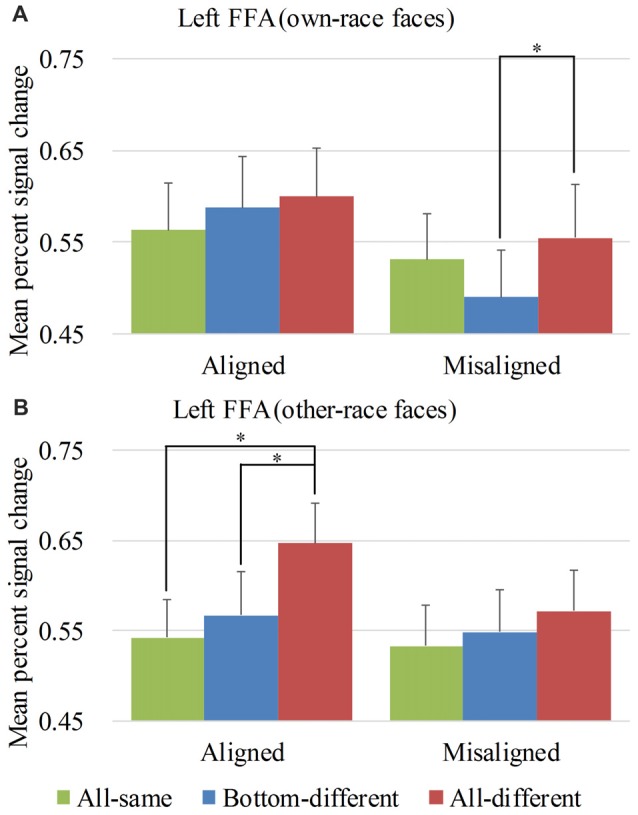
Mean PSC of the left FFA. Mean PSC of the left FFA elicited by each of the conditions during composite face recognition period for own-race faces **(A)** and other-race faces **(B)**. The error bars indicate the standard errors. The “*” indicates *p* < 0.017 (Bonferroni correction).

**Figure 6:**
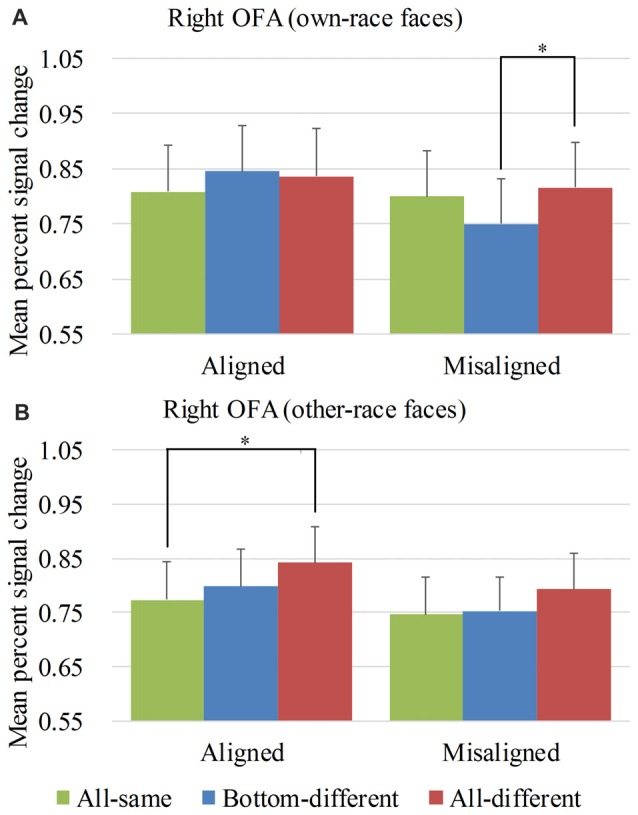
Mean PSC of the right occipital face areas (OFA). Mean PSC of the right OFA elicited by each of the conditions during composite face recognition period for own-race faces **(A)** and other-race faces **(B)**. The error bars indicate the standard errors. The “*” indicates *p* < 0.017 (Bonferroni correction).

**Figure 7:**
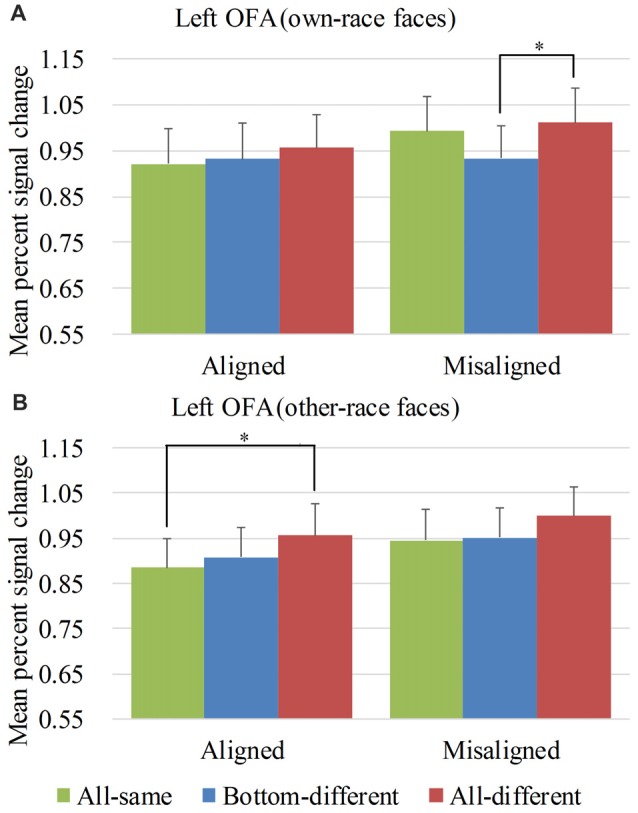
Mean PSC of the left OFA. Mean PSC of the left OFA elicited by each of the conditions during composite face recognition period for own-race faces **(A)** and other-race faces **(B)**. The error bars indicate the standard errors. The “*” indicates *p* < 0.017 (Bonferroni correction).

Four traditional cortical areas with preferential responses to faces were identified by the contrast of ([own- and other-race faces] minus non-face objects) using the data from the localizer sessions. These face preferential regions were the right FFA of 39 participants (MNI coordinates [mm]: 42 ± 3, −51 ± 6, −18 ± 3), the left FFA of 35 participants (MNI coordinates [mm]: −40 ± 3, −53 ± 6, −19 ± 3), the right OFA of 35 participants (MNI coordinates [mm]: 40 ± 5, −77 ± 5, −11 ± 4), and the left OFA of 31 participants (MNI coordinates [mm]: −40 ± 5, −79 ± 5, −11 ± 3). Supplementary Figure S2 showed each participant’s peak of ROIs. Supplementary Tables S1–S4 described the detailed information of each ROI. The loci of the face sensitive regions were consistent with those reported by previous studies (e.g., Kanwisher and Yovel, 2006; Fairhall and Ishai, 2007; Liu et al., 2015). We therefore defined these face preferential regions as the ROIs of the present study. Figures 4–7 show the PSCs of all ROIs, and Supplementary Figures S4–S7 show their individual dots plots, respectively.

#### ROI Analysis Results Within the Right FFA

Figure 4 showed the PSC of the right FFA elicited by 12 experimental conditions. A repeated-measure three-way ANOVA (*face race* × *alignment* × *identity*) performed on the PSCs revealed significant main effects of *identity* (*F*_(2,76)_ = 20.593, *p* < 0.05) and *alignment* (*F*_(1,38)_ = 46.526, *p* < 0.05). The main effect of *face race* was not significant, (*F*_(1,38)_ = 0.042, *p* = 0.839). These results paralleled the behavioral findings.

However, unlike the behavioral findings, the crucial interaction of *face race* × *alignment* × *identity* was significant (*F*_(2,76)_ = 4.814, *p* < 0.05). To explore the crucial effect of the interaction further, we performed analyses on the PSCs for own-race faces and other-race faces separately. For the PSC elicited by own-race faces, a repeated measure two-way ANOVA of (*alignment* × *identity*) revealed a significant interaction of *alignment* × *identity* (*F*_(2,76)_ = 9.683, *p* < 0.05), as well as significant main effects of *identity* (*F*_(2,76)_ = 9.988, *p* < 0.05) and *alignment* (*F*_(1,38)_ = 43.930, *p* < 0.05). As shown in Figure 4A, there was a significant difference among the three levels (i.e., *all-same*, *bottom-different* and *all-different*) for both *aligned* (*F*_(2,76)_ = 5.603, *p* < 0.05), and *misaligned* (*F*_(2,76)_ = 13.201, *p* < 0.05). More specifically, *all-different* engendered significantly more PSC than *all-same* for both *aligned* (*t*_(38)_ = 2.627, *p* < 0.017 [Bonferroni correction]), and *misaligned* (*t*_(38)_ = 3.948, *p* < 0.017 [Bonferroni correction]). These results replicated the robust face adaptation effect whereby the same face presented sequentially leads significantly less activations in the FFA when compared with the condition where two different faces are presented sequentially. However, we found no significant differences in these response decreases between *aligned* and *misaligned* (*t*_(38)_ = −0.441, *p* = 0.622). These findings suggested the equal sizes of releases of adaptation for both *aligned* and *misaligned*.

In contrast, *bottom-different* engendered significantly greater activations than *all-same* only for *aligned* (*t*_(38)_ = 3.393, *p* < 0.017 [Bonferroni correction]) but not for *misaligned* (*t*_(38)_ = −1.724, *p* = 0.093). Further, the PSC difference of the *bottom-different* minus *all-same* for *aligned* trials was greater than that for the *misaligned* (*t*_(38)_ = 4.110, *p* < 0.017 [Bonferroni correction]), resulting in a significant interaction of *identity* (*all-same* vs. *bottom-different*) × *alignment* (*aligned* vs. *misaligned*; *F*_(1,38)_ = 16.890, *p* < 0.017 [Bonferroni correction]). More importantly, for *aligned*, the PSC difference of the *bottom-different* minus *all-same* trials was equal to the difference of the *all-different* minus *all-same* trials (i.e., the normal release of adaptation; *t*_(38)_ = −0.373, *p* = 0.711). However, for *misaligned*, this contrast was significant (*t*_(38)_ = −4.661, *p* < 0.017 [Bonferroni correction]) due to the fact that the PSC for *all-different* was significantly larger than that for *bottom-different* (*t*_(38)_ = 4.661, *p* < 0.017 [Bonferroni correction]). These findings met the criteria of neural composite face effect. They suggested that, for *aligned*, although the top halves of the faces of *bottom-different* are the same, they were perceived to have different identities by participants such that they, like the faces with completely different identities (i.e., *all-different*), lead to a normal release of adaptation. However, this effect disappeared when the top and bottom halves of faces were misaligned (i.e., *misaligned*). This evidence suggested that the right FFA presented a neural composite face effect for own-race faces.

As for the PSC elicited by other-race faces, a repeated measure two-way ANOVA of (*alignment* × *identity*) only revealed significant main effects of *identity* (*F*_(2,76)_ = 13.977, *p* < 0.05) and *alignment* (*F*_(1,38)_ = 24.017, *p* < 0.05). The interaction of *alignment* × *identity* was not significant (*F*_(2,76)_ = 1.112, *p* = 0.334). As shown in Figure 4B, there were significant or marginally significant difference among the three levels (i.e., *all-same*, *bottom-different* and *all-different*) for both *aligned* (*F*_(2,76)_ = 15.032, *p* < 0.05) and *misaligned* (*F*_(2,76)_ = 2.717, *p* = 0.072), respectively. More specifically, the PSC for *all-different* was greater than those for *all-same* for both *aligned* (*t*_(38)_ = 4.900, *p* < 0.017 [Bonferroni correction]) and *misaligned* (*t*_(38)_ = 2.996, *p* < 0.017 [Bonferroni correction]). There was no significant difference in such increase in PSCs between *aligned* and *misaligned* (*t*_(38)_ = 1.782, *p* = 0.083), suggesting an equal magnitude of releases of adaptation for both *aligned* and *misaligned*. This release of adaptation was similar to that of own-race faces and suggested that the fMRI responses of the right FFA were also sensitive to the change of identities of other-race faces. However, in contrast to the PSC elicited by own-race faces, there was no significant difference in PSCs of other-race faces between *bottom-different* and *all-same* regardless of whether the trials were *aligned* (*t*_(38)_ = 0.663, *p* = 0.511) or *misaligned* (*t*_(38)_ = 0.039, *p* = 0.969). This finding did not meet the criteria of the neural composite effect above mentioned. It should be noted that, for *aligned* other-race faces, *all-different* was greater than *bottom-different* (*t*_(38)_ = 5.014, *p* < 0.017 [Bonferroni correction]). This finding suggested that *all-different* elicited release of adaptation of fMRI responses relative to both *all-same* and *bottom-different*. These findings altogether suggested that within the right FFA, for *aligned* other-race faces, the *bottom-different* faces were not perceived as the pair of faces with different identities. Thus, there was not any neural composite face effect for other-race faces within the right FFA.

As the holistic processing of faces can be broken down by the misalignment between the top and bottom haves of faces, some studies referred the decrease in behavior performance of *aligned* *bottom-different* faces relative to *misaligned* *bottom-different* face as behavior composite face effect (for review, see Rossion, 2013). Michel et al. (2006) found that such decrease in holistic processing of faces was greater for own-race faces than for other-race faces, suggesting a stronger behavior composite face effect for own-race faces than for other-race-faces. Similar to this analysis, we performed a 2 × 2 repeated-measure two-way ANOVA, namely *face race* (*own-race* vs. *other-race*) by *alignment* (*aligned* vs. *misaligned*) on the PSCs of the four main conditions for the *bottom-different* level of *identity* to further investigate the difference in the composite face effect associated with face races. The interaction of *face race* × *alignment* was significant (*F*_(1,38)_ = 6.791, *p* < 0.017 [Bonferroni correction]). *Post hoc* tests revealed that the increase in PSC of the right FFA elicited by* the aligned* minus *misaligned* trials was significant for both own-race faces (*t*_(38)_ = 7.391, *p* < 0.017 [Bonferroni correction]) and other-race faces (*t*_(38)_ = 2.528, *p* < 0.017 [Bonferroni correction]). Further, such increase in PSC was greater for own-race faces than for other-race faces (*t*_(38)_ = 2.606, *p* < 0.017 [Bonferroni correction]). In contrast, such interaction was not significant for the other levels of *identity* (*F*_(1,38)_ = 0.045, *p* = 0.832 for *all-same* and *F*_(1,38)_ = 3.349, *p* = 0.075 for *all-different*), paralleling to the findings of Michel et al. (2006).

Additionally, to further investigate the relation between the behavior performance and the fMRI responses in the present study, we first correlated the differences in accuracy rate between *aligned* faces and *misaligned* faces to the difference in PSC of the same contrast at each level of the *identity* separately for own-race faces and other-race faces. However, none of significant correlation coefficients was observed (*p*s > 0.017 [Bonferroni correction]).

We repeated performing this correlation analyses by replacing accurate rate with correct RT at each level of the *identity* separately for own-race faces and other-race faces. For own-race face, only at the *bottom-different* level, the difference in RT of *aligned* minus *misaligned* significantly positively correlated with the difference in PSC of the same contrast (*r* = 0.516, *p* < 0.017 [Bonferroni correction]). However, for other-race faces at the same level (i.e., the *bottom-different* level), this correlation analysis revealed no significant relation (*r* = 0.004, *p* = 0.980). In other words, only for own-race faces, the more the recognition of the top haves of faces was disrupted by the bottom haves of faces (e.g., the prolonged RT), the more it elicited the release of adaptation of fMRI responses. In contrast, for each of other two levels of identity (e.g., *all-same* and *all-different*) of each of own-race faces and other-race faces, the same correlation analysis revealed none significant relation (*ps* > 0.05). Together, these findings suggested that own-race faces produced a stronger neural composite face effect than other-race faces in the right FFA.

#### ROI Analysis Results Within the Left FFA

Figure 5 showed the PSC of the left FFA elicited by 12 experimental conditions. A repeated-measure three-way ANOVA (*face race* × *alignment* × *identity*) performed on the PSCs revealed a significant main effect of *identity* (*F*_(2,68)_ = 7.111, *p* < 0.05) and *alignment* (*F*_(1,34)_ = 15.259, *p* < 0.05). However, neither the interaction of *face race* × *alignment* × *identity* (*F*_(2,68)_ = 2.900, *p* = 0.062) nor the main effect of the *face race* (*F*_(1,34)_ = 0.280, *p* = 0.600) was significant.

To explore the response patterns resulting from own-race faces and other-race faces, paralleling the right FFA analysis, we performed analyses on the PSCs elicited by own-race faces and other-race faces separately. For the PSC elicited by own-race faces, a repeated measure two-way ANOVA of (*alignment* × *identity*) revealed a significant interaction of *alignment* × *identity* (*F*_(2,68)_ = 3.315, *p* < 0.05), as well as a main effect of *alignment* (*F*_(1,34)_ = 16.442, *p* < 0.05). The main effect of *identity* was not significant (*F*_(2,68)_ = 1.838, *p* = 0.167). As shown in the Figure 5A, there was a significant difference among the three levels (i.e., *all-same*, *bottom-different*, and *all-different*) for *misaligned* (*F*_(2,68)_ = 3.701, *p* < 0.05) but not for *aligned* (*F*_(2,68)_ = 1.018, *p* = 0.367). More specifically, own-race faces elicited significant PSC increase for *all-different* relative to *bottom-different* only for *misaligned* (*t*_(34)_ = 2.729, *p* < 0.017 [Bonferroni correction]) but not for *aligned* (*t*_(34)_ = 0.476, *p* = 0.637). However, the PSC difference of the *all-different* minus *bottom-different* trials for *misaligned* was not significantly different from that for *aligned* (*t*_(34)_ = 1.844, *p* = 0.074). In contrast to the PSC elicited by own-race faces within the right FFA, there was no significant difference in PSCs of own-race faces within the left FFA between *bottom-different* and *all-same* regardless of whether the trials were *aligned* (*t*_(34)_ = 1.046, *p* = 0.303) or *misaligned* (*t*_(34)_ = −1.940, *p* = 0.061). In addition, there was no significant difference between *all-different* and *all-same* regardless of whether the trials were *aligned* (*t*_(34)_ = 1.232, *p* = 0.227) or *misaligned* (*t*_(34)_ = 0.873, *p* = 0.389). These response patterns did not meet the criteria of neural composite face effect, suggesting that, for own-race faces, there was not any composite face effect in the fMRI responses in the left FFA.

As for the PSC elicited by other-race faces, a repeated measure two-way ANOVA of (*alignment* × *identity*) only revealed a significant main effect of *identity* (*F*_(2,68)_ = 7.625, *p* < 0.05). Neither the interaction of *alignment* × *identity* (*F*_(2,68)_ = 2.119, *p* = 0.128) nor the main effect of* alignment* (*F*_(1,34)_ = 3.704, *p* = 0.063) was significant. As shown in Figure 5B, there was a significant difference among the three levels (i.e., *all-same*, *bottom-different*, and *all-different*) for *aligned* (*F*_(2,68)_ = 7.723, *p* < 0.05) but not for *misaligned* (*F*_(2,68)_ = 1.457, *p* = 0.240). More specifically, the activation for *all-different* was significantly greater than that for *all-same* for *aligned* (*t*_(34)_ = 4.218, *p* < 0.017 [Bonferroni correction]) but not for *misaligned* (*t*_(34)_ = 2.136, *p* > 0.017 [Bonferroni correction]). However, the PSC difference of the *all-different* minus *all-same* for *aligned* trials was not significantly different from that for *misaligned* (*t*_(34)_ = 2.252, *p* > 0.017 [Bonferroni correction]). Additionally, the PSC for *all-different* was greater than that for *bottom-different* for *aligned* (*t*_(34)_ = 2.818, *p* < 0.017 [Bonferroni correction]) but not for *misaligned* (*t*_(34)_ = 0.902, *p* = 0.373). However, PSC difference of the *all-different* minus *bottom-different* trials for *aligned* was not significantly different from that for *misaligned* (*t*_(34)_ = 1.491, *p* = 0.145). In addition, there was no significant difference between *bottom-different* and *all-same* regardless of whether the trials were *aligned* (*t*_(34)_ = 0.814, *p* = 0.422) or *misaligned* (*t*_(34)_ = 0.647, *p* = 0.522). These response patterns did not meet the criteria of neural composite face effect, suggesting that, for other-race faces, there was not any composite face effect in the fMRI responses in the left FFA. We also performed repeated-measure two-way ANOVAs of *face race* × *alignment* on the activation of the left FFA for each of level of *identity*. However, none of their interactions of *face race* × *alignment* was significant (*p*s > 0.017 [Bonferroni correction]).

An interesting finding should be noted: although neither own-race faces nor other-race faces produced the neural composite face effect in the left FFA, the *aligned* other-race faces elicited identical response patterns in the right FFA (Figure 4B left) and the left FFA (Figure 5B left). In contrast, the response patterns for the *aligned* own-race faces in the right FFA (Figure 4A left) and left FFA (Figure 5A left) were different. This finding suggested that the right and left FFAs processed the *aligned* own-race faces differently whereas they processed the other-race faces similarly.

#### ROI Analysis Results Within the Right OFA

Figure 6 showed the PSC of the right OFA elicited by the 12 experimental conditions. A repeated-measure three-way ANOVA (*face race* × *alignment* × *identity*) performed on the PSCs revealed a significant main effect of *identity* (*F*_(2,68)_ = 4.652, *p* < 0.05) and *alignment* (*F*_(1,34)_ = 11.169, *p* < 0.05). However, neither the interaction of *face race* × *alignment* × *identity* (*F*_(2,68)_ = 1.850, *p* = 0.165) nor the main effect of the *face race* (*F*_(1,34)_ = 0.464, *p* = 0.500) was significant.

To explore the response patterns resulting from own-race faces and other-race faces, we performed analyses on the PSCs elicited by own-race faces and other-race faces separately.

For the PSC elicited by own-race faces, a repeated measure two-way ANOVA of (*alignment* × *identity*) revealed a significant interaction of *alignment* × *identity* (*F*_(2,68)_ = 5.050, *p* < 0.05), as well as a main effect of *alignment* (*F*_(1,34)_ = 8.193, *p* < 0.05). The main effect of *identity* was not significant (*F*_(2,68)_ = 1.816, *p* = 0.170). As shown in the Figure 6A, there was a significant difference among the three levels (i.e., *all-same*, *bottom-different* and *all-different*) for *misaligned* (*F*_(2,68)_ = 4.973, *p* < 0.05) but not for *aligned* (*F*_(2,68)_ = 1.619, *p* = 0.206). More specifically, own-race faces elicited significant PSC increase for *all-different* relative to *bottom-different* only for *misaligned* (*t*_(34)_ = 2.914, *p* < 0.017 [Bonferroni correction]) but not for *aligned* (*t*_(34)_ = −0.446, *p* = 0.659). Further, this PSC difference of the *all-different* minus *bottom-different* for *misaligned* was significantly greater than that for the *aligned* (*t*_(34)_ = 2.613, *p* < 0.017 [Bonferroni correction]), resulting in a significant interaction of *identity* (*all-different* vs. *bottom-different*) × *alignment* (*aligned* vs. *misaligned*; *F*_(1,34)_ = 6.827, *p* < 0.017 [Bonferroni correction]). There was no significant difference in PSCs of own-race faces between *bottom-different* and *all-same* regardless of whether the trials were *aligned* (*t*_(34)_ = 1.607, *p* = 0.117) or *misaligned* (*t*_(34)_ = −2.424, *p* > 0.017 [Bonferroni correction]). In addition, there was no significant difference between *all-different* and *all-same* regardless of whether the trials were *aligned* (*t*_(34)_ = 1.354, *p* = 0.185) or *misaligned* (*t*_(34)_ = 0.722, *p* = 0.475).

As for the PSC elicited by other-race faces, a repeated measure two-way ANOVA of (*alignment* × *identity*) revealed the significant main effects of *alignment* (*F*_(1,34)_ = 6.306, *p* < 0.05) and *identity* (*F*_(2,68)_ = 3.983, *p* < 0.05). However, the interaction of *alignment* × *identity* was not significant (*F*_(2,68)_ = 0.242, *p* = 0.786). As shown in Figure 6B, there was a significant difference among the three levels (i.e., *all-same*, *bottom-different*, and *all-different*) for *aligned* (*F*_(2,68)_ = 3.411, *p* < 0.05) but not for *misaligned* (*F*_(2,68)_ = 1.554, *p* = 0.219). More specifically, the activation for *all-different* was significantly greater than that for *all-same* for *aligned* (*t*_(34)_ = 2.907, *p* < 0.017 [Bonferroni correction]) but not for *misaligned* (*t*_(34)_ = 1.638, *p* = 0.111). However, the PSC difference of *all-different* minus *all-same* for *aligned* trials was not significantly different from that for *misaligned* (*t*_(34)_ = 0.670, *p* = 0.508). There was no significant difference between *bottom-different* and *all-same* regardless of whether the trials were *aligned* (*t*_(34)_ = 0.841, *p* = 0.406) or *misaligned* (*t*_(34)_ = 0.274, *p* = 0.786).

Overall, in the right OFA, neither the response pattern of own-race faces nor that of other-race face met the criteria of neural composite face effect, and therefore there was no evidence of neural composite face effect in this ROI.

We also performed repeated-measure two-way ANOVAs of *face race* × *alignment* on the PSCs of the right OFA for each level of *identity*. None of their interactions of *face race* × *alignment* was significant (*p*s > 0.017 [Bonferroni correction]).

#### ROI Analysis Results Within the Left OFA

Figure 7 showed the PSC of the left OFA elicited by the 12 experimental conditions. A repeated-measure three-way ANOVA (*face race* × *alignment* × *identity*) performed on the PSCs revealed a significant main effect of *identity* (*F*_(2,60)_ = 8.873, *p* < 0.05) and *alignment* (*F*_(1,30)_ = 9.938, *p* < 0.05). However, neither the interaction of *face race* × *alignment* × *identity* (*F*_(2,60)_ = 0.788, *p* = 0.459) nor the main effect of the *face race* (*F*_(1,30)_ = 0.251, *p* = 0.620) was significant.

To explore the response patterns resulting from own-race faces and other-race faces, we performed analyses on the PSCs elicited by own-race faces and other-race faces separately.

For the PSC elicited by own-race faces, a repeated measure two-way ANOVA of (*alignment* × *identity*) revealed the significant main effects of *alignment* (*F*_(1,30)_ = 7.636, *p* < 0.05) and *identity* (*F*_(2,60)_ = 4.290, *p* < 0.05). However, the interaction of *alignment* × *identity* was not significant (*F*_(2,60)_ = 2.194, *p* = 0.120). As shown in the Figure 7A, there was a significant difference among the three levels (i.e., *all-same*, *bottom-different*, and *all-different*) for *misaligned* (*F*_(2,60)_ = 6.194, *p* < 0.05) but not for *aligned* (*F*_(2,60)_ = 0.928, *p* = 0.401). More specifically, own-race faces elicited significant PSC increase for *all-different* relative to *bottom-different* only for *misaligned* (*t*_(30)_ = 3.686, *p* < 0.017 [Bonferroni correction]) but not for *aligned* (*t*_(30)_ = 0.914, *p* = 0.368). However, this PSC difference of the *all-different* minus *bottom-different* for *misaligned* trials was not significantly different from that for *aligned* (*t*_(30)_ = 1.420, *p* = 0.116). There was no significant difference in PSCs of own-race faces between *bottom-different* and *all-same* regardless of whether the trials were *aligned* (*t*_(30)_ = 0.410, *p* = 0.685) or *misaligned* (*t*_(30)_ = −2.185, *p* > 0.017 [Bonferroni correction]). In addition, there was no significant difference between *all-different* and *all-same* regardless of whether the trials were *aligned* (*t*_(30)_ = 1.311, *p* = 0.200) or *misaligned* (*t*_(30)_ = 0.919, *p* = 0.365).

As for the PSC elicited by other-race faces, a repeated measure two-way ANOVA of (*alignment* × *identity*) revealed the significant main effects of *alignment* (*F*_(1,30)_ = 6.924, *p* < 0.05) and *identity* (*F*_(2,60)_ = 6.349, *p* < 0.05). However, the interaction of *alignment* × *identity* was not significant (*F*_(2,60)_ = 0.174, *p* = 0.841). As shown in Figure 7B, there was a significant difference among the three levels (i.e., *all-same*, *bottom-different*, and *all-different*) both for *aligned* (*F*_(2,60)_ = 4.013, *p* < 0.05) and *misaligned* (*F*_(2,60)_ = 3.428, *p* < 0.05). More specifically, the activation for *all-different* was significantly greater than that for *all-same* for *aligned* (*t*_(30)_ = 3.088, *p* < 0.017 [Bonferroni correction]) but not for *misaligned* (*t*_(30)_ = 2.380, *p* > 0.017 [Bonferroni correction]). However, the PSC difference of the *all-different* minus *all-same* for *aligned* trials was not significantly different from that for *misaligned* (*t*_(30)_ = 0.534, *p* = 0.598). There was no significant difference between *bottom-different* and *all-same* regardless of whether the trials were *aligned* (*t*_(30)_ = 0.762, *p* = 0.452) or *misaligned* (*t*_(30)_ = 0.299, *p* = 0.767).

Overall, in the left OFA, neither the response pattern of own-race faces nor that of other-race faces met the criteria of neural composite face effect, and therefore there was not any neural composite face effect in this ROI.

An interesting finding was that *aligned* other-race faces elicited increased fMRI responses for *all-different* faces than for *all-same* faces in both bilateral OFA. This finding indicated that *aligned* other-race faces with different identities elicited a release of adaptation of fMRI responses. In other words, the bilateral OFA were both sensitive to the identities of other-race face but not to own-race faces.

We also performed repeated-measure two-way ANOVAs of *face race* × *alignment* on the PSCs of the left OFA for each of level of *identity*. None of their interactions of *race* × *alignment* was significant (*p*s > 0.017 [Bonferroni correction]).

## Discussion

The present study examined the response patterns of the face-preferential areas in the ventral occipitotemporal cortex elicited by own-race and other-race faces using the composite face paradigm. The most important finding of the present study was that the right FFA exhibited a neural composite face effect only for own-race faces, which was evidenced by a release of adaptation when the composite faces were aligned, whereas such an effect was not observed for other-race faces. We also found that this race-related difference in neural composite face effect was not the exact neural counterpart of the behavioral face composite effect.

Many fMRI studies have reported that sequentially viewing the face with the same identity leads to adaptation in the FFA where the neural responses to the face become smaller with increased viewing (e.g., Andrews and Ewbank, 2004; Winston et al., 2004; Loffler et al., 2005). These findings suggested that there may exist a population of neurons sensitive to face identities in the FFA (Andrews and Ewbank, 2004; Loffler et al., 2005). Consistent with these existing findings, we found that for the own-race composite face, when the top and bottom halves were aligned, the right FFA showed stronger responses to the pair of faces with the same top but different bottom halves (i.e., *bottom-different*) than those with both the same top and the same bottom halves (i.e., *all-same*). Further, such increase in neural responses produced by the faces with different bottom haves (i.e., *bottom-different*) was equal to that produced by the faces with completely different identities (i.e., *all-different*). These findings suggested that the faces with the same top halves but different bottom halves elicited similar releases of adaptation to that of the faces with completely different identities. In other words, these faces were treated as if they had different identities from each other.

Our findings are highly consistent with Schiltz et al. (2010) who found that, when the top and bottom halves of the faces were aligned, the pairs of faces with the same top halves but different bottom halves elicited a release of adaptation with the same size as that elicited by the pair of faces with different top and bottom halves. The most plausible explanation of these findings was that although the participants were instructed to focus on the top halves of faces, the discrimination of these two faces were influenced by the information of the bottom halves of faces when the top and bottom halves of the composite faces were aligned (Michel et al., 2006). As a result, the pair of faces with the same top halves but different bottom halves were processed as faces with completely different identities. However, when the top and bottom halves of the pair of faces were misaligned, this composite face effect disappeared. For the *misaligned* condition, the release of adaptation from the right FFA responses was produced by the pair of faces with completely different identities (i.e., *all-different*), not by the change of the bottom halves of the faces alone (i.e., *bottom-different*). These findings taken together suggested that facial identity information was processed holistically in the right FFA, and any change of local features of a face, even though outside the visual focus, can lead to an illusion of change of the identity of this face.

In contrast, we did not observe the neural composite face effect in the right FFA for other-race faces. More specifically, when the top and bottom halves of the composite faces were aligned, the release of the adaptation from the right FFA responses was produced only by the faces with completely different identities. Although this response pattern of the *aligned* other-race faces was highly different from that of the *aligned* own-race faces, it was very similar to that of the *misaligned* own-race faces. These findings suggested that, for other-race faces, even though their bottom halves were aligned to their respective top halves, the processing of the latter was not influenced by the former. Given the close relationship between the composite effect and facial holistic processing, our findings suggested that the right FFA might be less involved in the holistic processing of other-race faces than own-race ones. In contrast, the present study did not find the neural composite face effect for either own-race faces or other-race faces within the left FFA, suggesting that the left FFA might not be involved in holistic processing of faces.

Thus, for own-race faces, the neural composite effect was different in the right vs. left FFAs. However, this was not the case for other-race faces. For other-race faces, though they did not produce the neural composite face effect within either the right FFA or the left FFA, they elicited similar response patterns for these two brain regions. In other words, within each of the bilateral FFA, for *aligned* other-race faces, the adaptation release of the fMRI responses was only produced by the pair of faces with completely different identities (i.e., *all-different*), independent of the change of the bottom halves of the faces. This finding suggested that both the right FFA and the left FFA were sensitive to the change of identities of other-races faces, and therefore may be equally involved in the processing of other-race faces. Thus, the right and left FFAs might serve similar processing functions for other-race faces, but different functions for own-race faces.

Why were the neural composite face effects of own-race faces different in the right vs. left FFA? Extensive research has shown that own-race faces produce greater activations in the right hemisphere than the left hemisphere, more specifically the right FFA (Kanwisher and Yovel, 2006). Further, recent studies suggested that the right hemisphere was more involved in holistic/configural processing whereas the left hemisphere was more involved in the processing of the local/featural processing (e.g., Dien, 2009; Cattaneo et al., 2014). More specifically, Schiltz et al. (2010) found that the holistic processing of faces was more involved in the right FFA than the left FFA. Additionally, a recent study reported that the right FFA was closely associated with the processing of low frequency information, whereas the left FFA was recruited by high frequency information processing (Woodhead et al., 2011). The processing of the holistic facial information that is reflected by the composite face effect is dependent greatly on the processing of low frequency (Goffaux and Rossion, 2006). These findings taken together thus support our hypotheses that own-race faces are processed more holistically than other-race faces.

The present study failed to find any neural composite face effects in the bilateral OFA. This may be accounted for by the role that the OFA plays in the neural network of face processing (Liu et al., 2010). According to the network theory of face processing, there is a hierarchical pathway of face processing which extends from the primary visual cortex to the high cognitive brain regions (Haxby et al., 2000; Fairhall and Ishai, 2007). In this network, the OFA is involved in the processing of local features of faces and then transmit this information to the FFA (in particular the right FFA). In contrast, the right FFA integrates the local features along with the second-order relations between them into a gestalt and thereby produces a holistic representation of a face (Haxby et al., 2000; Goffaux and Rossion, 2006; Fairhall and Ishai, 2007; Liu et al., 2010; Schiltz et al., 2010). Thus, the right FFA, but not the OFA, is a crucial node in the network of face processing that transmits the holistic information of a face to different higher level functional brain regions (e.g., the temporal pole, the prefrontal cortex and amygdala) to extract multi-dimensional facial information (e.g., identity, sematic and emotion). It should be noted that the fMRI responses of the bilateral OFA were both sensitive to the change of the identities for other-race faces but not for own-race faces. Given the important role of the OFA in processing local features of faces, this finding suggested that the recognition of other-race faces may be more dependent on local features than that of own-race faces. Thus, the present findings regarding OFAs and FFAs for own- vs. other-race faces taken together suggested that along the pathway of the bottom-up face processing, own-race faces and other-race faces presented the holistic processing difference as early as when they were processed in the right FFA.

The present fMRI study failed to find the difference in behavior composite face effect between own-race faces and other-race faces, although an additional behavior experiment out of fMRI scanner using the similar experimental procedure as the fMRI experiment of the present study revealed this difference (for more detail see Part IV of Supplementary Materials). The absence of the race-related behavior difference for fMRI study may be potentially due to multiple factors such as the change in the experimental procedure of the present study from that of Michel et al. (2006), and the specific visual and auditory environment of the MRI scanner.

In addition, this finding may suggest that the composite effect based on the behavior records and that based on the right FFA responses may not necessarily be the same thing. In the present study, the *bottom-different* trials of the *aligned* own-race faces as a whole led to a significant increase in error rate, and at the same time resulted in the release of adaptation in the fMRI response of the right FFA. However, in terms of a single trial, it was not necessarily that error recognition led to the release of adaptation of the fMRI response of the right FFA and vice versa. More importantly, according to the theory of network of face processing (Haxby et al., 2000; Fairhall and Ishai, 2007), the behavior performance in face recognition is the integration of all stages of face processing from the visual cortex to the frontal lobe, and therefore is supported by the whole brain network rather than one specific brain region such as the right FFA. In other words, although the right FFA plays a core role in the face processing, it is not sufficient for the successful face recognition and therefore need the help of other brain regions. As demonstrated in the present study, *aligned* own-race faces elicited distinctive response patterns for the right FFA and the left FFA, whereas *aligned* other-race faces elicited identical response patterns for the right FFA and the left FFA. Additionally, in the present study, the bilateral OFA was sensitive to the identities for other-race faces but not for own-race faces. This suggested that, compared to the processing own-race faces, the processing of other-race faces may be more compensated by other brain regions for the insufficiency in the face recognition of the right FFA.

The disagreement between the response of the right FFA and behavior performance was also frequently observed in recent studies about covert face processing. For example, Kouider et al. (2009) found that although the initial priming faces were not consciously perceived by participants (thus without any behavioral indications), it can still produce an adaptation effect. Lehmann et al. (2004) reported that the right FFA responded more to faces that had been previously seen (i.e., old faces) than to new faces regardless of whether the faces were remembered or forgotten. However, it showed no response differences between the remembered old faces and forgotten old faces. Simon et al. (2011) also reported a prosopagnosia patient who did not recognize the previously familiar faces but presented increased FFA responses to these faces relative to the unknown faces. These previous studies suggested that the behavior performance might be dissociated from the responses of the right FFA.

These existing studies along with the findings of the present study suggested that the response pattern of the separate right FFA should not be construed as the actual neural counterpart of the behavioral composite effect. These findings also imply that in the future studies of the relationship between behavior performance and brain regions, a multi-variate analysis including multiple brain regions is more suitable than the investigation of only one brain region (e.g., the right FFA).

One limitation of the present study was that we only tested Chinses participants. One may argue that the difference in the neural composite face effect found in the present study might be due to differences in low-level properties between the faces of the two races rather than the race difference itself. However, several approaches we had taken to mitigate this issue. First, as described in “Stimuli and Experiment Procedures” section, the low-level visual properties of the images of the present study such as luminance and contrast had been adjusted to be balanced using a standard MATLAB procedure (SHINE, Willenbockel et al., 2010). Further, all face images were converted into gray-scale images and the external features of each face (e.g., ears and hair style) were removed. Moreover, the composited face images of the present study (about 80%) had been used in a previous behavior study (Ge et al., 2009) that found cross-race effects in face recognition for both Caucasian participants and Chinese participants. Finally, there was few of difference in the face configural information between own-race faces and other-race faces (for more detail see Part I of Supplementary Materials). Additionally, our participants were recruited from a city where 94% of the population is Han Chinese. The participants reported to have no prior direct contact with other-race individuals. Thus, the neural ORE found in the present study was likely due to the fact that the participants had more experience with own-race Chinese faces than other-race Caucasian faces. However, to confirm this possibility fully, one needs to replicate the present study with Chinese and Caucasian participants with limited exposure to Caucasian or Chinese individuals, respectively.

Another limitation of the present study was that the own-race faces and other-race faces were included different fMRI runs. Our original aim was to avoid the signal overlapping of the own-race faces and other-race faces, and therefore compare the response patterns related to these two races of faces. However, this led to a possibility that the race-related difference in the fMRI responses that we expected may result from the inter-run difference. Although the present study counterbalanced the order of the own-race faces runs and other-race faces runs, and the GLM included an inter-run normalization, these methods may not completely remove this inter-run effect.

## Conclusion

The present studies used fMRI methodology and the composite face paradigm. We examined the response patterns of the face-preferential cortical areas in the ventral occipitotemporal cortex (i.e., the bilateral FFAs and the bilateral OFAs) elicited by own-race faces and other-race faces. Our most important finding was that the right FFA exhibited a neural composite face effect for own-race faces in terms of a release of adaptation but not for other-race faces, with the absence of the race-related difference in behavior composite face effect. These findings suggest that the right FFA is more involved in holistic processing of individual own-race faces as opposed to other-race faces. They also suggested that the neural composite effect observed in the right FFA may not be the exact neural counterpart of the behavioral face composite effect. The findings of the present study revealed that, along the pathway of the bottom-up face processing, own-race faces and other-race faces presented the holistic processing difference as early as when they were processed in the right FFA.

## Author Contributions

NX, JL, HL and KL: experimental design and data recording. GZ and JL: experimental data analysis. GZ, JL and KL: manuscript writing. GZ, JL, SJW and KL: manuscript revision.

## Conflict of Interest Statement

The authors declare that the research was conducted in the absence of any commercial or financial relationships that could be construed as a potential conflict of interest.

## Footnotes

^1^http://www.fil.ion.ucl.ac.uk/spm
