# Supplementary material for: The Fusiform Face Area Plays a Greater Role in Holistic Processing for Own-Race Faces Than Other-Race Faces
Source: Front Hum Neurosci. 2018 Jun 1;12:220. doi: 10.3389/fnhum.2018.00220 (PMC5992462; doi:10.3389/fnhum.2018.00220)
Supplement: Supplementary file 1 [file Presentation_1.PDF]

frontiers

Supplementary Materials

The Fusiform Face Area Plays a Greater Role in Holistic Processing for Own-Race Faces than Other-Race Faces

Guifei Zhou[1], Jiangang Liu[1*], Naiqi G. Xiao[2], Si Jia Wu[3], Hong Li[4,5,6*], Kang Lee[3,7*]

[1]School of Computer and Information Technology, Beijing Jiaotong University, Beijing, China

[2]Department of Psychology, Princeton University, Princeton, New Jersey 08544, USA

[3]Dr. Eric Jackman Institute of Child Study, University of Toronto, Toronto, Ontario M5R 2X2, Canada

[4]College of Psychology and Sociology, Shenzhen University, Shenzhen, China

[5]Center for Language and Brain, Shenzhen Institute of Neuroscience, Shenzhen 518057, China

[6]Shenzhen Key Laboratory of Affective and Social Cognitive Science, Shenzhen University, Shenzhen, China

[7]Department of Psychology, Zhejiang Normal University, Jinhua, China

*** Correspondence:**
Jiangang Liu
liujg@bjtu.edu.cn

Hong Li
lihongszu@szu.edu.cn

Kang Lee
kang.lee@utoronto.ca

1 Part I. Face visage analysis

1.1 Method

The key points of faces stimuli were exacted using FaceTrack package in Visage|SDK (Pandzic and Forchheimer, 2003, Visage Technologies AB, http://visagetechnologies.com/products-and-services/visagesdk/).

First, these key facial points were used to measure the configural information among eyes, nose and mouth in each aligned face (Mondloch et al., 2002; Tanaka et al., 2014; Wang et al., 2015). We referred (1) distance between two pupils as to eyes spacing (Supplementary Figure 1 d1, between point 1 and 2), (2) distance between the tip of nose and the middle of mouth as to mouth spacing (Supplementary Figure 1 d2, between point 3 and 4), (3) and distance between the tip of nose and the line between two pupils as to nose spacing (Supplementary Figure 1 d3, between point 3 and 5). Additionally, we measured the horizontal width of each face using distance between the right and left margin of outline (Supplementary Figure 1 d4, between point 6 and 7), and the vertical height of each

face using distance between the chin and the line between the right and left margin of outline (Supplementary Figure 1 d5, between point 8 and 9). For each face image, the eyes spacing (i.e., d1) was normalized to the horizontal width (i.e., d4), whereas the mouth spacing (i.e., d2) and the nose spacing (i.e., d3) were normalized to the vertical height (i.e., d5). Then, for each type of spacing (e.g., d1), a two-sample t test was used to explore whether there was difference between own-race faces and other-race faces across all faces. We performed these t-tests separately for male faces and female faces.

1.2 Results

There was not any significant difference in the eyes spacing between own-race faces and other-race faces for either female (t (46) = 0.849, p = 0.400) faces or male faces (t (46) = -0.614, p = 0.542). There were also no any significant differences in mouth spacing between own-race faces and other-race faces for either female (t(46) = 1.761, p = 0.085) faces or male faces (t(46) = -0.948, p = 0.348). As for nose spacing, the difference between own-race faces and other-race faces was significant only for female faces (t (46) = 2.497, p = 0.016), but not for male faces (t (46) = 1.960, p = 0.056).

In summary, for male faces, none of three types of spacing showed significant difference between own-race faces and other-race faces. For female faces, only nose spacing showed significant difference between own-race faces and other-race faces. To explore whether this gender-related difference led to the difference in behavior performance between male faces and female faces, a paired t test was used to examine whether there was difference in the participants' behavior performance (i.e., accuracy rate or correct response time) between female faces and male faces for each of 12 experiment conditions respectively. We found no significant differences between participates' behavior performance for female faces and male faces in any of experiment conditions (ps > 0.05).

1.3 Discussion

These findings suggested that there was few difference in configural information between own-race faces and other-race faces used in the present study. Additionally, the faces used in the present study have been balanced in their low-level visual properties. Further, they have been demonstrated to lead to behavior other-race effect for Chinese participants as well as Caucasian participant (Ge et al., 2009). Thus, it was very likely that the race-related difference in neural composite face effect of the present study resulted from the difference of own-face faces vs. other-race faces rather than differences in any of Chinese faces vs. Caucasian faces.

1.4 Figure

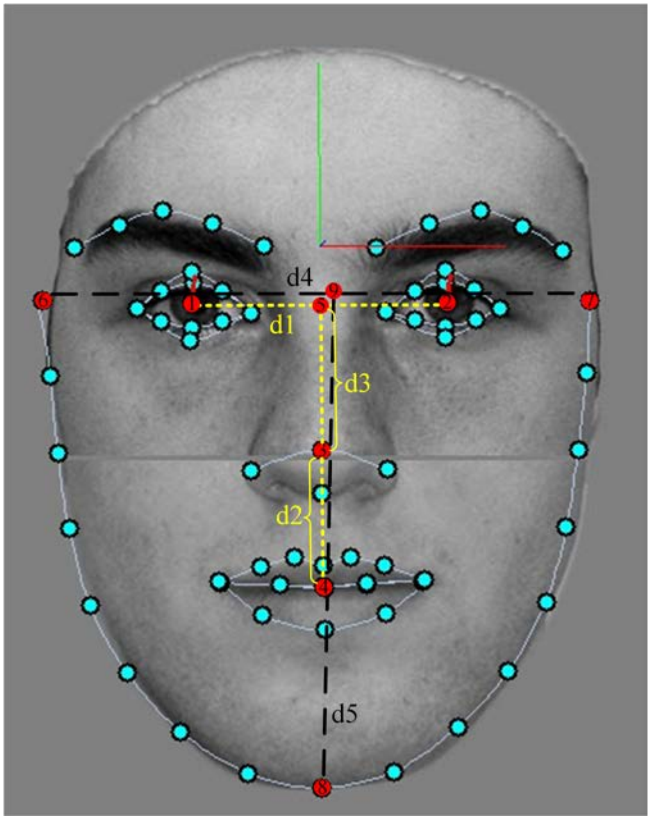

Supplementary Figure 1. The illustration of visage points of a face. Visage point 1 and 2 indicated the right and the left pupil, respectively; Visage point 3 indicated the tip of the nose; Visage point 4 indicated the middle of the mouth; Visage point 5 indicated the foot of the perpendicular drawn from tip of nose to the line between two pupils; Visage point 6 and 7 indicated the right and left margin of face outline, respectively; Visage point 8 indicated the chin; Visage point 9 indicated the foot of a perpendicular draw from the chin to the line between the right and left margin of face outline. d1 indicated the distance between the point 1 and point 2; d2 indicated the distance between point 3 and point 4; d3 indicated the distance between point 3 and point 5; d4 indicated the distance between the point 6 and point 7; d5 indicated the distance between point 8 and point 9.

2 Part II. Individual locations of all ROIs

2.1 Figure

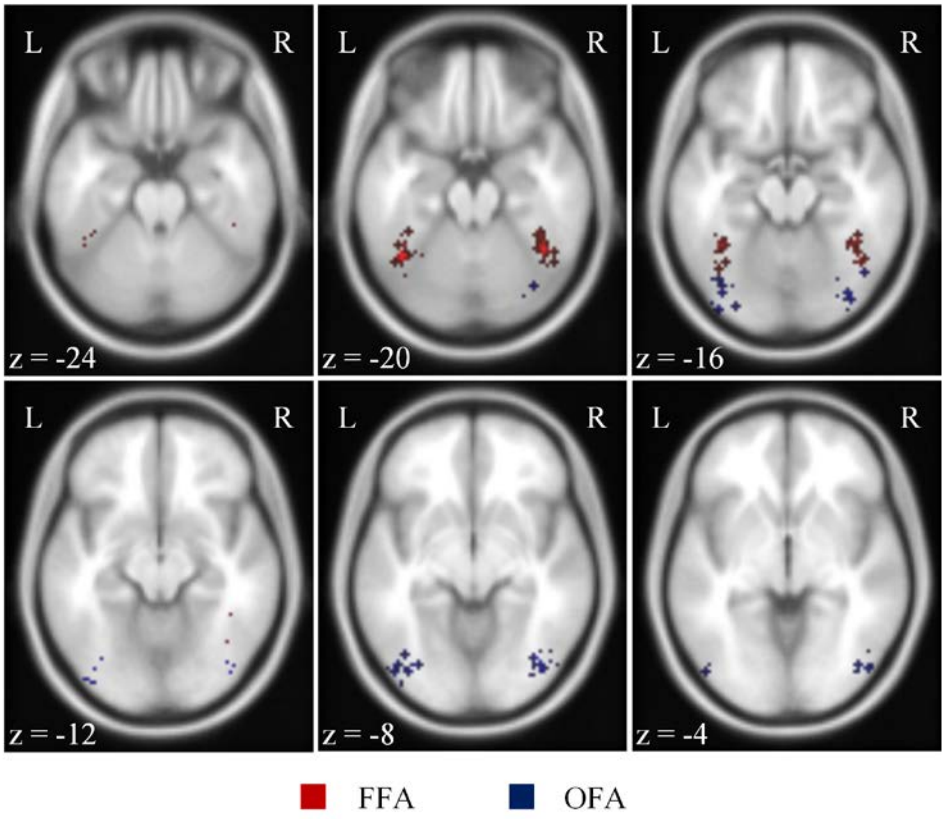

Supplementary Figure 2. Each participant's peak of ROIs. Axial slices showing the peak voxels of FFA (red) and OFA (blue) of each individual participant on an MNI template brain.

2.2 Tables

Supplementary Table 1. Detailed information of right FFA for each subject. The "—" indicates not applied.

Subject No.	Cluster voxels	MNI coordinates			Contrast value (t)	Threshold (p)	Radius (mm)
		x	y	z			
1	302	42	-54	-20	13.41	0.0001	—
2	67	44	-62	-18	6.06	0.0001	—
3	179	38	-50	-18	10.77	0.0001	14
4	371	42	-52	-20	17.38	0.0001	—
5	168	42	-46	-22	8.13	0.0001	—
6	116	42	-54	-16	7.31	0.0010	—
7	310	38	-48	-16	13.55	0.0001	—
8	69	38	-44	-20	4.50	0.0010	—
9	46	40	-48	-14	7.83	0.0001	—
10	149	44	-54	-14	13.14	0.0001	—
11	183	44	-54	-20	8.63	0.0001	—
12	43	44	-60	-16	6.88	0.0001	5
13	227	46	-58	-16	11.54	0.0001	—
14	309	42	-48	-14	15.15	0.0001	13
15	120	44	-48	-18	7.13	0.0001	11
16	31	42	-58	-22	5.41	0.0001	5
17	64	40	-54	-14	7.89	0.0001	—
18	455	40	-52	-20	8.90	0.0001	—
19	10	44	-38	-24	4.01	0.0001	—
20	145	40	-46	-22	8.92	0.0001	—
21	12	48	-60	-20	3.72	0.0010	—
22	333	42	-44	-22	19.57	0.0001	—
23	259	40	-42	-20	11.84	0.0001	—
24	—	—	—	—	—	—	—
25	72	42	-46	-12	8.61	0.0001	—
26	47	42	-56	-18	6.95	0.0001	—
27	12	48	-54	-20	3.57	0.0050	—
28	61	40	-50	-16	8.32	0.0001	—
29	284	42	-50	-20	8.44	0.0001	—
30	103	40	-62	-12	7.01	0.0001	7
31	84	40	-60	-18	8.11	0.0001	—
32	185	42	-52	-20	13.22	0.0001	—
33	175	42	-46	-14	13.16	0.0001	—
34	284	36	-50	-14	13.45	0.0001	—
35	399	38	-54	-22	12.54	0.0001	—
36	152	42	-44	-16	11.22	0.0001	—
37	76	42	-48	-18	9.18	0.0001	—
38	178	40	-48	-20	13.74	0.0001	—
39	370	42	-60	-18	15.49	0.0001	12
40	44	36	-52	-18	5.80	0.0001	—

Supplementary Table 2. Detailed information of left FFA for each subject. The “—” indicates not applied.

Subject No.	Cluster voxels	MNI coordinates			Contrast value (t)	Threshold (p)	Radius (mm)
		x	y	z			
1	146	-42	-58	-20	9.46	0.0001	—
2	78	-36	-60	-16	8.24	0.0001	—
3	120	-36	-46	-14	11.96	0.0001	—
4	43	-40	-50	-18	9.06	0.0001	—
5	70	-42	-56	-20	8.22	0.0001	—
6	19	-38	-52	-16	4.88	0.0050	—
7	159	-40	-64	-16	6.88	0.0001	—
8	79	-42	-56	-20	5.95	0.0010	—
9	—	—	—	—	—	—	—
10	77	-42	-54	-18	10.36	0.0001	—
11	23	-34	-54	-22	5.69	0.0001	—
12	119	-40	-52	-18	11.06	0.0001	—
13	24	-40	-44	-24	4.36	0.0010	—
14	—	—	—	—	—	—	—
15	190	-42	-60	-14	10.21	0.0001	—
16	107	-42	-62	-22	8.22	0.0001	7
17	—	—	—	—	—	—	—
18	433	-42	-44	-18	10.40	0.0001	—
19	14	-44	-46	-24	3.79	0.0050	—
20	171	-44	-50	-24	7.28	0.0001	—
21	12	-46	-60	-20	5.02	0.0001	—
22	102	-40	-50	-18	11.35	0.0001	—
23	14	-42	-56	-20	4.18	0.0001	—
24	—	—	—	—	—	—	—
25	—	—	—	—	—	—	—
26	82	-40	-50	-22	11.94	0.0001	—
27	38	-40	-50	-14	7.60	0.0001	—
28	18	-40	-48	-18	4.36	0.0010	—
29	278	-42	-58	-20	13.09	0.0001	—
30	20	-40	-68	-18	3.20	0.0050	—
31	134	-40	-54	-18	6.73	0.0001	—
32	21	-38	-48	-16	7.41	0.0001	—
33	80	-38	-42	-20	7.51	0.0001	—
34	186	-36	-50	-16	12.93	0.0001	—
35	75	-38	-58	-22	5.32	0.0001	—
36	40	-38	-42	-24	8.55	0.0001	—
37	70	-30	-56	-22	3.82	0.0050	—
38	76	-38	-56	-20	8.48	0.0001	—
39	339	-44	-50	-20	11.77	0.0001	14
40	15	-40	-56	-22	3.56	0.0100	—

Supplementary Table 3. Detailed information of right OFA for each subject. The “—” indicates not applied.

Subject No.	Cluster voxels	MNI coordinates			Contrast value (t)	Threshold (p)	Radius (mm)
		x	y	z			
1	213	46	-78	-2	16.20	0.0001	12
2	233	34	-82	-10	6.47	0.0001	—
3	338	36	-78	-16	10.07	0.0001	14
4	154	38	-80	-14	5.43	0.0001	—
5	13	36	-72	-8	4.32	0.0001	—
6	—	—	—	—	—	—	—
7	42	40	-74	-10	4.67	0.0001	—
8	—	—	—	—	—	—	—
9	—	—	—	—	—	—	—
10	12	38	-72	-10	4.03	0.0050	—
11	42	36	-88	-14	5.51	0.0001	—
12	53	40	-68	-10	7.00	0.0001	5
13	20	42	-78	-8	5.88	0.0001	—
14	423	40	-74	-12	10.27	0.0001	13
15	212	46	-68	-10	7.92	0.0001	11
16	46	46	-66	-16	7.03	0.0001	5
17	—	—	—	—	—	—	—
18	195	44	-76	-12	4.01	0.0001	—
19	10	34	-82	-14	4.33	0.0001	—
20	163	30	-80	-18	10.99	0.0001	—
21	12	42	-80	-6	3.16	0.0050	—
22	145	30	-70	-14	13.06	0.0001	—
23	177	48	-78	-4	5.65	0.0001	—
24	—	—	—	—	—	—	—
25	57	36	-74	-20	7.08	0.0001	—
26	17	36	-82	-10	5.42	0.0001	—
27	55	40	-76	-8	6.76	0.0001	—
28	68	42	-76	-4	6.47	0.0001	—
29	42	38	-84	-14	3.36	0.0050	—
30	51	36	-76	-10	3.62	0.0010	7
31	15	50	-76	-6	4.37	0.0010	—
32	51	38	-82	-16	4.15	0.0010	15
33	45	36	-78	-14	6.22	0.0001	—
34	19	42	-80	-12	3.64	0.0050	—
35	183	36	-78	-10	7.99	0.0001	—
36	28	48	-68	-2	7.67	0.0001	—
37	512	38	-72	-10	7.97	0.0001	—
38	80	46	-74	-10	6.28	0.0001	—
39	137	40	-80	-6	5.77	0.0001	12
40	17	38	-80	-14	3.92	0.0050	—

Supplementary Table 4. Detailed information of left OFA for each subject. The “—” indicates not applied.

Subject No.	Cluster voxels	MNI coordinates			Contrast value (t)	Threshold (p)	Radius (mm)
		x	y	z			
1	147	-38	-86	-12	9.34	0.0001	—
2	84	-36	-76	-10	6.97	0.0001	—
3	172	-44	-76	-8	10.08	0.0001	—
4	45	-46	-80	-8	5.37	0.0001	—
5	14	-40	-86	-14	5.02	0.0001	—
6	—	—	—	—	—	—	—
7	—	—	—	—	—	—	—
8	30	-38	-80	-14	4.39	0.0010	—
9	—	—	—	—	—	—	—
10	—	—	—	—	—	—	—
11	10	-40	-88	-16	4.73	0.0050	—
12	91	-46	-82	-10	4.78	0.0001	—
13	18	-40	-70	-16	4.29	0.0050	—
14	13	-44	-84	-14	3.86	0.0050	—
15	—	—	—	—	—	—	—
16	68	-38	-74	-16	6.62	0.0001	7
17	—	—	—	—	—	—	—
18	468	-30	-86	-16	6.21	0.0001	—
19	38	-40	-86	-12	5.60	0.0001	—
20	180	-44	-70	-14	7.17	0.0001	—
21	12	-44	-84	-12	4.58	0.0001	—
22	309	-34	-76	-10	17.97	0.0001	—
23	14	-46	-76	-6	4.78	0.0001	—
24	—	—	—	—	—	—	—
25	15	-50	-78	-10	5.10	0.0001	—
26	31	-42	-84	-12	3.60	0.0010	—
27	49	-42	-86	-10	6.13	0.0001	—
28	17	-48	-80	-4	5.14	0.0010	—
29	12	-40	-70	-8	4.99	0.0001	—
30	—	—	—	—	—	—	—
31	43	-38	-78	-12	7.68	0.0001	—
32	14	-42	-88	-10	5.53	0.0050	—
33	24	-36	-80	-10	5.53	0.0001	—
34	16	-32	-74	-14	6.05	0.0001	—
35	21	-38	-80	-8	4.90	0.0010	—
36	15	-32	-76	-8	5.18	0.0010	—
37	15	-40	-78	-10	3.36	0.0050	—
38	60	-34	-72	-12	10.97	0.0001	—
39	183	-38	-78	-16	7.06	0.0001	14
40	—	—	—	—	—	—	—

3 Part III. Individual dots plots of behavior performance and PSCs of all ROIs

3.1 Figures

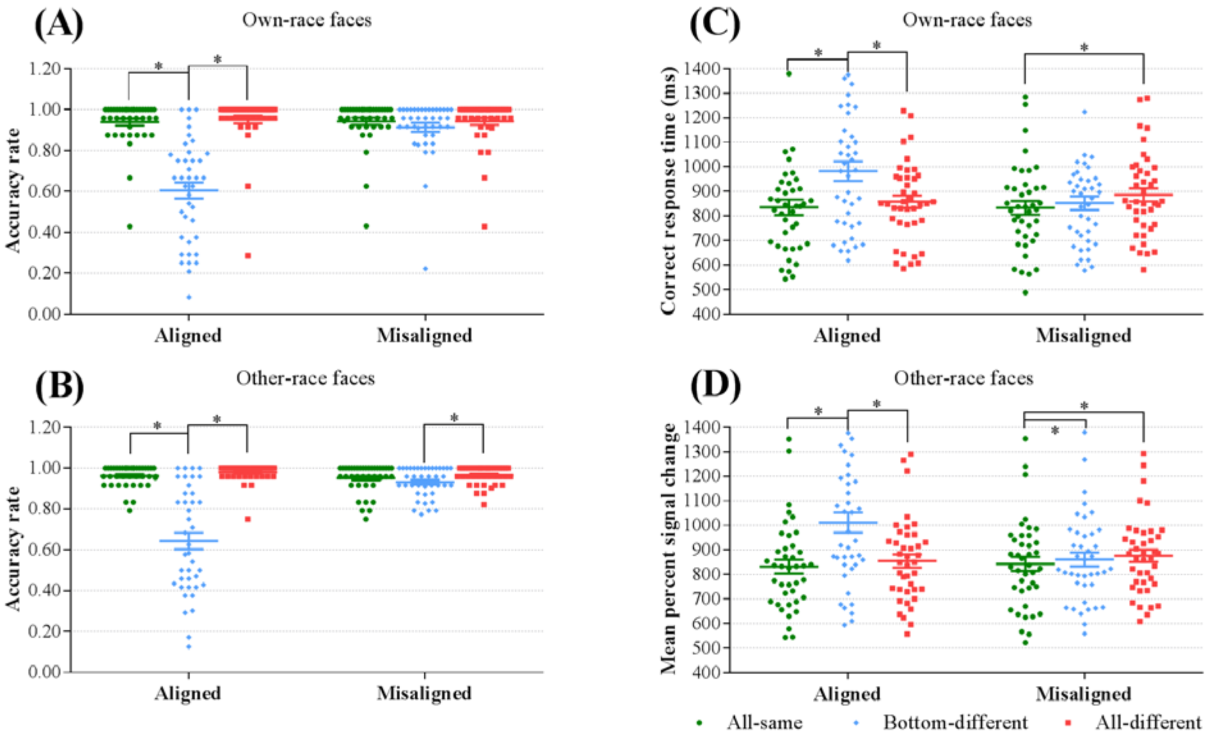

Supplementary Figure 3. Each participant's behavior performance. Figure showed the mean accuracy rates of own-race faces (A) and other-race faces (B) as well as the mean correct response time of own-race faces (C) and other-race faces (D). The error bars indicate the standard errors. The "*" indicates $p < 0.017$ (Bonferroni correction).

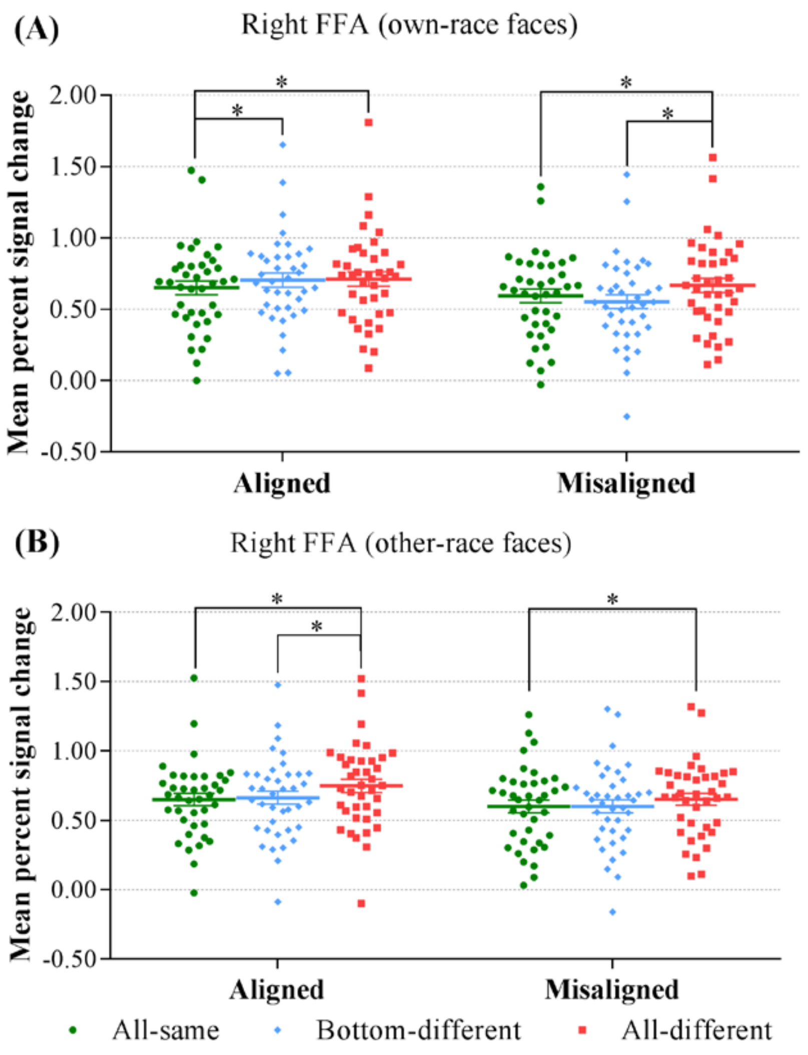

Supplementary Figure 4. Each participant's mean percent signal change of the right FFA. Mean percent signal change of the right FFA elicited by each of the conditions during composite face recognition period for own-race faces (A) and other-race faces (B). The error bars indicate the standard errors. All the statistical significant levels have been Bonferroni corrected. The "*" indicates $p < 0.017$ (Bonferroni correction).

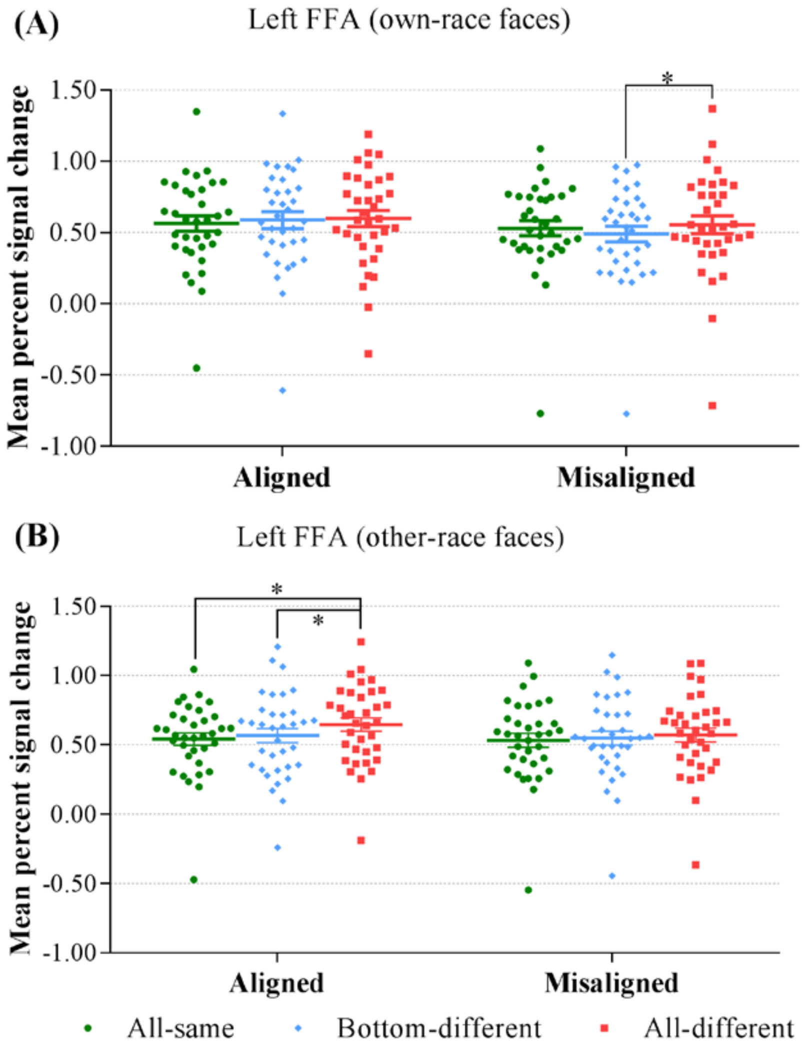

Supplementary Figure 5. Each participant's mean percent signal change of the left FFA. Mean percent signal change of the left FFA elicited by each of the conditions during composite face recognition period for own-race faces (A) and other-race faces (B). The error bars indicate the standard errors. All the statistical significant levels have been Bonferroni corrected. The "*" indicates $p < 0.017$ (Bonferroni correction).

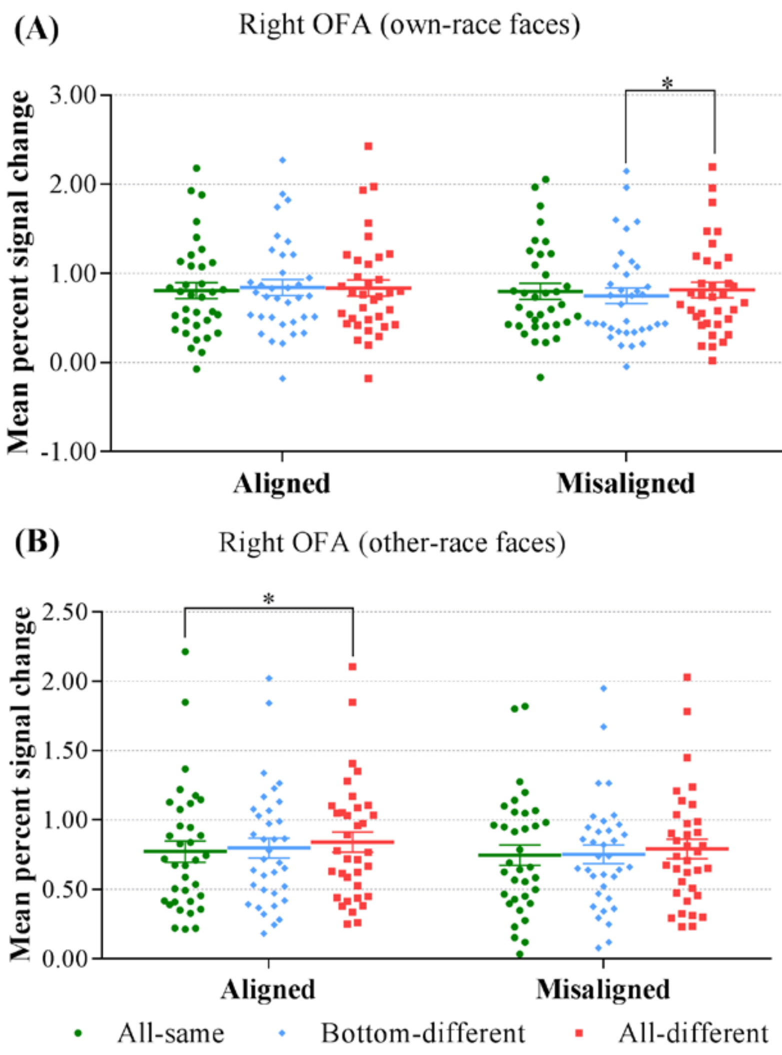

Supplementary Figure 6. Each participant's mean percent signal change of the right OFA. Mean percent signal change of the right OFA elicited by each of the conditions during composite face recognition period for own-race faces (A) and other-race faces (B). The error bars indicate the standard errors. The "*" indicates $p < 0.017$ (Bonferroni correction).

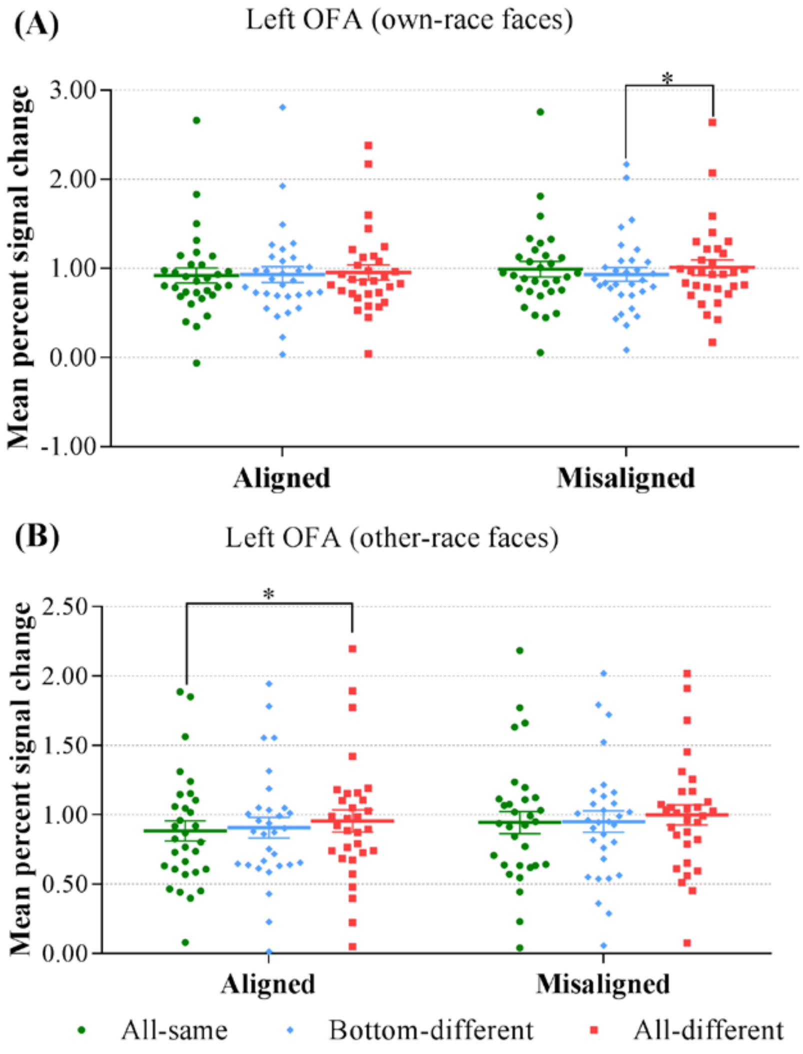

Supplementary Figure 7. Each participant's mean percent signal change of the left OFA. Mean percent signal change of the left OFA elicited by each of the conditions during composite face recognition period for own-race faces (A) and other-race faces (B). The error bars indicate the standard errors. The "*" indicates $p < 0.017$ (Bonferroni correction).

4 Part IV. Advance behavior experiment

4.1 Participants

Twenty-five healthy, right-handed Han Chinese adults (17 males; From 18 to 21 years old) with normal or corrected-to-normal vision participated. These participants were not included in the fMRI experiment of the present study. As reported by these participants, none of them had the experience of ever working or living with Caucasians, or being engaged into the work that needed to directly contact with Caucasians or Caucasian face images. A written informed consent was signed by all

participants prior to the experiment. The present study was approved by the Southwest University research ethics committee.

4.2 Stimuli and experimental procedures

The behavior experiment used the same face stimuli and also the same 2 × 2 × 3 factorial within-participant design as the composite face recognition period of the fMRI experiment of the present study.

Each participate performed 288 trials, one half of which were Chinese face trials and the other half were Caucasian face trials. The Chinese face trials and the Caucasian face trials were randomly presented. Each Chinese face trial began with a red fixation as a prompt (500 ms), and then included a pair of faces presented in sequence (3000 ms for the first face, and 150 ms for the second face) with an interval of a blank screen of 500 ms between them, and ended with the instruction presentation of 2000 ms. When the instruction was presented, the participants were required to focus on the top halves of these two faces, and to make a decision as to whether they were the same or different via button presses (right and left buttons for the "same" or "different" responses counterbalanced across participants). These 144 pairs of faces consisted of a 2 × 3 factorial within-participant design (24 trials for each cell), namely *alignment* (*aligned* vs. *misaligned*) by *identity* (*all-same* vs. *bottom-different* vs. *all-different*). The experimental design of the Caucasian face trials was the same as that of the Chinese face trials except that the Chinese faces were replaced by Caucasian faces.

4.3 Results

The three-way ANOVA analysis (*face race* × *alignment* × *identity*) performed on the correct response time revealed a significant interaction of *face race* × *alignment* × *identity* (F (2, 48) = 4.175, $p < 0.05$). Additionally, the main effects of *face race* (F (1, 24) = 7.039, $p < 0.05$), *alignment* (F (1, 24) = 12.195, $p < 0.05$), and *identity* (F (2, 48) = 10.816, $p < 0.05$) were all significant.

Further, paralleling Michel et al., (2006), we performed a two-way ANOVA analysis on the four conditions of the *bottom-different* level of *identity* for the correct response time. As shown in Supplementary Figure 8, the main effect of *face race* (F (1, 24) = 14.929, $p < 0.05$) and that of *alignment* (F (1, 24) = 21.293, $p < 0.05$) are both significant. The latter is due to the fact that the participants performed slower for *aligned* faces than for *misaligned* faces regardless of own-race faces and other-race faces (t (24) = 4.614, $p < 0.05$), suggesting a composite face effect. More importantly, the two-way ANOVA analysis also revealed a significant interaction effect of *face race* × *alignment* (F (1, 24) = 4.692, $p < 0.05$). Subsequent t-test revealed the increase in response time of *aligned* trials minus *misaligned* trials (i.e., the behavior composite effect [Michel et al., 2006]) was significant for own-race faces (t (24) = 4.345, $p < 0.05$) and other-race faces (t (24) = 2.991, $p < 0.05$). However the composite effect was larger for own-race than for other-race faces (t (24) = 2.166, $p < 0.05$), and therefore led to the significant interaction effect of *face race* × *alignment*. This finding suggested that behavior composite effect was stronger for own-race faces than for other-race faces, which was greatly consistent with findings of Michel et al. (2006).

In addition, we also separately performed a two-way ANOVA of (*face race* × *alignment*) for the correct response time at the other two level of *identity*, namely *all-different* and *all-same*. For *all-different* level, there was no significant interaction effect of *face race* × *alignment* (F (1, 24) = 0.021, $p = 0.887$) or main effect of *face race* (F (1, 24) = 1.594, $p = 0.219$) or main effect of *alignment* (F (1,

24) = 0.798, p = 0.381). Similarly, for *all-same* level, there was no significant interaction effect of *face race* × *alignment* (F (1, 24) = 1.262, p = 0.272) or main effect of *face race* (F (1, 24) = 0.006, p = 0.939) or main effect of *alignment* (F (1, 24) = 1.222, p = 0.280).

As the distribution of the accuracy rate is binomial rather than normal, a generalized estimating equations analysis instead of ANOVA was performed on the accuracy rate with the *face race* (*own-race* vs. *other-race*), *alignment* (*aligned* vs. *misaligned*), and *identity* (*all-same* vs. *bottom-different* vs. *all-different*) as the within-subject variables, and the binomial response with a logit link function as the response. The generalized estimating equations analysis (*face race* × *alignment* × *identity*) did not reveal significant interaction effect (*Wald* x^2 (2) = 1.757, p = 0.415) or main effect of *face race* (*Wald* x^2 (1) = 0.171, p = 0.679) or main effects of *alignment* (*Wald* x^2 (1) = 2.554, p = 0.110). However, the main effect of *identity* (*Wald* x^2 (2) = 119.605, $p < 0.05$) was significant. Paralleling Michel et al., (2006), we performed a two-way ANOVA analysis on the four conditions of the *bottom-different* level of *identity* for the accuracy (see Supplementary Figure 9). Only the main effect of *alignment* was significant (*Wald* x^2 (1) = 46.816, $p < 0.05$) due to the fact that participants showed less accuracy for *aligned* trials than for *misaligned* trails regardless of own-race faces and other-race faces ($p < 0.05$), suggesting a composite face effect. However, neither the main effect of *face race* (*Wald* x^2 (1) = 0.385, p = 0.535) nor the interaction effect of *face race* × *alignment* (*Wald* x^2 (1) = 0.373, p = 0.542) was significant.

In addition, we also separately performed the generalized estimating equations analysis of (*face race* × *alignment*) for the accuracy rate at the other two level of *identity*, namely *all-different* and *all-same*. For *all-different* level, only the main effect of *alignment* was significant (*Wald* x^2 (1) = 8.173, $p <$ 0.05) due to the fact that the participants recognized faces more accurately for *aligned* faces than for *misaligned* faces ($p < 0.05$). However, neither the main effect of *face race* (*Wald* x^2 (1) = 0.720, p = 0.396) nor the interaction effect of *face race* × *alignment* (*Wald* x^2 (1) = 0.028, p = 0.867) was significant. As for *all-same* level, there was no significant interaction effect of *face race* × *alignment* (*Wald* x^2 (1) = 1.045, p = 0.307) or main effect of *face race* (*Wald* x^2 (1) = 0.831, p = 0.362) or main effect of *alignment* (*Wald* x^2 (1) = 2.054, p = 0.152) was significant.

4.4 Figures

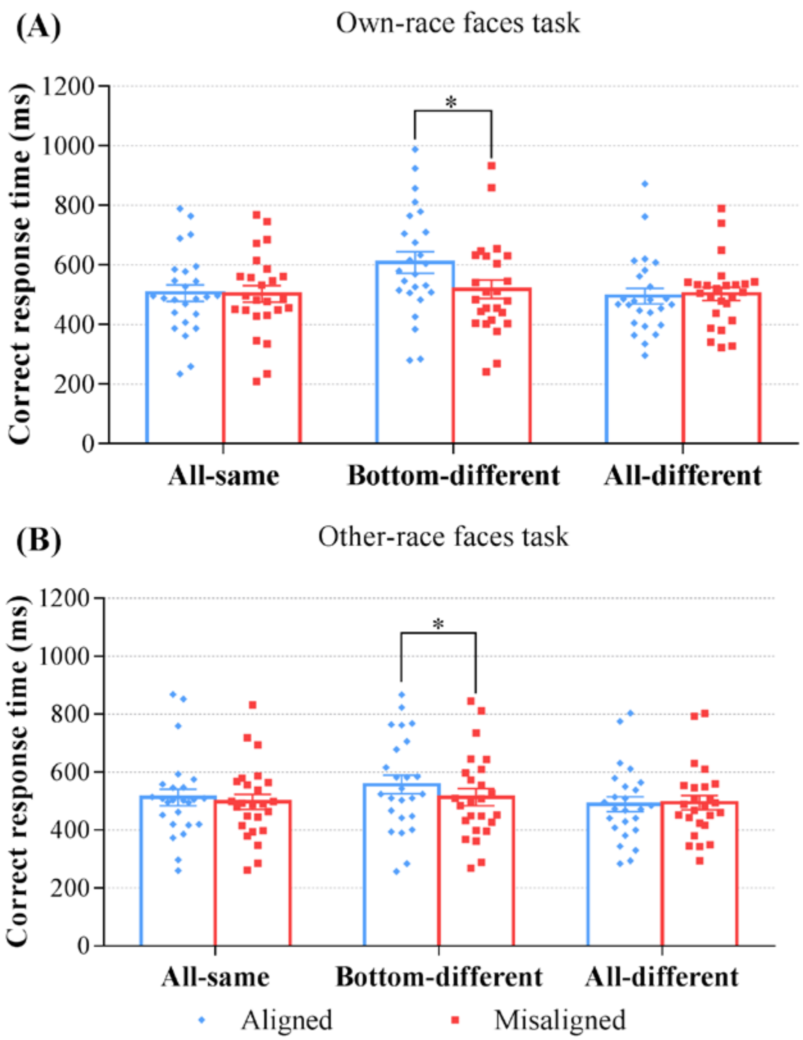

Supplementary Figure 8. Correct response time. The mean correct response time of own-race faces task (A) and other-race faces task (B) were calculated through all participants. The error bars indicate the standard errors. The "*" indicates $p < 0.05$.

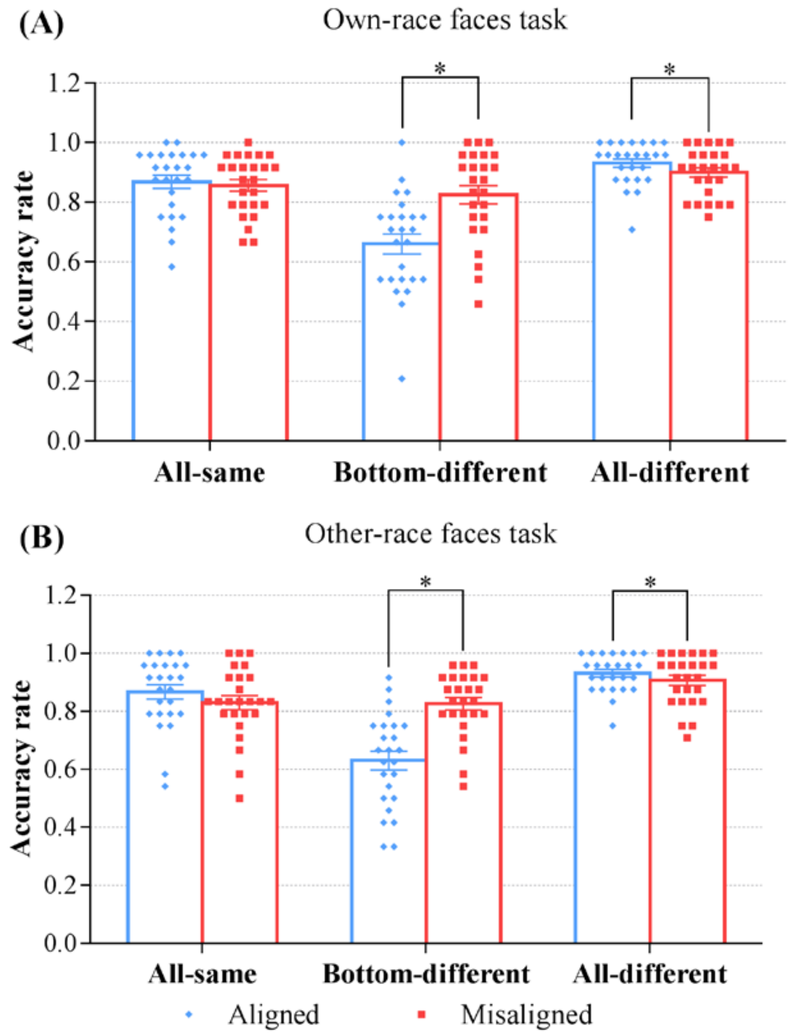

Supplementary Figure 9. Accuracy rates. The mean accuracy rates of own-race faces task (A) and other-race faces task (B) were calculated through all participants. The error bars indicate the standard errors. The "*" indicates $p < 0.05$.
